# Observational Dose-Response Meta-Analysis Methods May Bias Risk Estimates at Low Consumption Levels: The Case of Meat and Colorectal Cancer

**DOI:** 10.1016/j.advnut.2024.100214

**Published:** 2024-03-21

**Authors:** Jane G Pouzou, Francisco J Zagmutt

**Affiliations:** EpiX Analytics, LLC. Fort Collins, CO, United States

**Keywords:** model uncertainty, red and processed meat, carcinogenicity, confounding and bias in dose-response models, model assumptions in nutrition

## Abstract

Observational studies of foods and health are susceptible to bias, particularly from confounding between diet and other lifestyle factors. Common methods for deriving dose-response meta-analysis (DRMA) may contribute to biased or overly certain risk estimates. We used DRMA models to evaluate the empirical evidence for colorectal cancer (CRC) association with unprocessed red meat (RM) and processed meats (PM), and the consistency of this association for low and high consumers under different modeling assumptions. Using the Global Burden of Disease project’s systematic reviews as a start, we compiled a data set of studies of PM with 29 cohorts contributing 23,522,676 person-years and of 23 cohorts for RM totaling 17,259,839 person-years. We fitted DRMA models to lower consumers only [consumption < United States median of PM (21 g/d) or RM (56 g/d)] and compared them with DRMA models using all consumers. To investigate impacts of model selection, we compared classical DRMA models against an empirical model for both lower consumers only and for all consumers. Finally, we assessed if the type of reference consumer (nonconsumer or mixed consumer/nonconsumer) influenced a meta-analysis of the lowest consumption arm. We found no significant association with consumption of 50 g/d RM using an empirical fit with lower consumption (relative risk [RR] 0.93 (0.8–1.02) or all consumption levels (1.04 (0.99–1.10)), while classical models showed RRs as high as 1.09 (1.00–1.18) at 50g/day. PM consumption of 20 g/d was not associated with CRC (1.01 (0.87–1.18)) when using lower consumer data, regardless of model choice. Using all consumption data resulted in association with CRC at 20g/day of PM for the empirical models (1.07 (1.02–1.12)) and with as little as 1g/day for classical models. The empirical DRMA showed nonlinear, nonmonotonic relationships for PM and RM. Nonconsumer reference groups did not affect RM (*P* = 0.056) or PM (*P* = 0.937) association with CRC in lowest consumption arms. In conclusion, classical DRMA model assumptions and inclusion of higher consumption levels influence the association between CRC and low RM and PM consumption. Furthermore, a no-risk limit of 0 g/d consumption of RM and PM is inconsistent with the evidence.

**Statements of significance**

This article describes critical issues in classical methods of observational dose-response modeling that may lead to overestimates of risk at low levels of consumption. We propose alternative methods which more accurately reflect the uncertainty in dose-response meta-analytic models and show that risk overestimation at low consumption can result from modeling assumptions and from the influence of higher consumption amounts. Unprocessed and processed meat and colorectal cancer are used to demonstrate methods for dose-response that can be adapted to other observational evidence of dose-dependent risk and could be used in developing dietary guidelines in a transparent and systematic way.

## Introduction

Dose-response meta-analysis (DRMA) is an important tool to summarize evidence for nutritional epidemiology and burden of disease studies. Numerous DRMA studies on foods and noncommunicable health outcomes have been published [[1], [2], [3]], largely using the methods established by Greenland and Longnecker [4]. This approach allows for the estimation of dose-dependent effect sizes, typically relative risks (RRs), between exposure and health outcomes while considering correlations in exposure groups within studies. Just as with traditional meta-analysis, DRMA models typically reduce the variance in the RR as the evidence between studies is pooled. The resulting increase in precision is beneficial when the studies are consistent in exposure type and when potential confounding variables are controlled for, as in models of experimental exposure to chemical and microbial hazards where the concept of dose-response (DR) models were first developed and applied [5,6]. In these experimental DR models, dose is typically a single chemical or microbial hazard (rather than a complex food), assigned in a randomized design and delivered in controlled amounts, minimizing potential confounding and bias. In contrast, the observational studies of diet and noncommunicable disease used for nutritional DRMA are based on cohorts from heterogeneous populations and diets, and thus known to be susceptible to biases and confounding [7,8]. These limitations include measurement bias, particularly of lifetime consumption [[9], [10], [11]] and residual confounding [12] with other diet components and lifestyle covariates [13,14]. Their combination may sometimes result in predictable biases in the DRMA [[15], [16], [17]].

For example, when there is a casual association between consumption and outcome, if the reference group for the RR estimates contains individuals who are occasional consumers of the foods of interest (as opposed to individuals who never consume the food), the association will be underestimated [18]. Systematic underestimation of the highest consumption group via some imputation methods might, on the contrary, overestimate the food-outcome association [19]. Random error and conflicting biases in study sources may cancel these effects or even reverse them, making such systematic errors difficult to identify in the final DRMA [20]. If the shrinkage of the error from DRMA methods, intended to increase the precision of an RR and food consumption estimate, is combined with bias in the estimations because of confounding or other causes as described above, an artificially certain and biased effect estimate will ensue [21].

Model choices may also influence the overall conclusions of a meta-analysis. Models commonly used in DRMA models combine studies via a “2-stage” approach. In brief, a DR model of the same functional form (e.g., linear) is estimated for each study, and the coefficients of these models are then combined with a meta-analysis, weighted by the studies’ SE [22]. This method assumes that 1 underlying DR is true for each individual study and at all consumption levels. An alternative “point-wise” method, proposed by Crippa et al. [23], applies the best-fitting model to each study individually and then uses the weighed prediction of RRs from each study rather than combining their coefficients. This method may permit the per-study models to more closely represent each data set—an extension of the proposed benefit of the 2-stage model as described by Berlin et al. [22]. This approach can also be restricted to only the RRs for consumption amounts in each study, avoiding spurious extrapolations. This may result in combined DRs that better represent the empirical results from observational studies.

A case with a large body of observational literature with inconsistent results, and even conflicting results between different meta-analyses, is the relationship between unprocessed red meat (RM) and processed meat (PM) and colorectal cancer (CRC). Some meta-analyses report strong observational and mechanistic DR associations [24], whereas others report no or weak associations [25,26]. Consumption assessments in the observational studies used for meta-analysis are of varying specificity (meaning some include all meats or PM and RM in 1 category and some do not specify if PMs are included with RMs or not), confounding variables controlled for are highly variable, and the reference group may or may not include meat consumers. Furthermore, the range of consumptions may differ considerably. For example, in the Oba et al. [27] study set in Takayama, Japan, the highest consumption group median was 56.6 g/d, but in the Pietinen et al. [28] study of Finnish males, the highest consumption group median was 99 g/d. DRMA methods that can accurately capture the heterogeneity in these studies are key to understanding the association of RM and PM and CRC and therefore providing sound dietary recommendations, particularly at the lower amounts consumed by most people.

The aim of this study was to use DRMA models to evaluate the empirical evidence for CRC association with RM and PM. We first examine the DR association observed at lower consumption of RM and PM and contrast this result with DR derived from data at all consumption levels to test whether the association is equivalent for low- and high-meat consumers. We further evaluate how model selection impacts these inferences by comparing commonly applied linear and spline-based approaches with a more novel empiric method. Lastly, we examine whether the presence of RM or PM consumers in the study’s reference group affects the DR association.

## Methods

### Overall approach

The Global Burden of Disease Project (GBD) regularly estimates the burden of illnesses and deaths attributable to various causes, and their association with risk factors including dietary components. The purpose of these publications is to estimate the current population’s illness burden associated with, in the case of dietary risks, the consumption of a specific food, based on current consumption and DRMA estimated from systematic review results [29,25]. As such, systematic reviews on the association between dietary factors such as RM and PM intake and health impacts such as CRC are regularly updated [29,30], and the citations used are reported in the Global Health Data Exchange (GHDx) [31]. The design and execution of these systematic reviews are consistent with PRISMA guidelines [32]. We therefore constructed an initial data set based on the citations for health impacts from consumption of RM and PM reported in GHDx. We further identified studies with only nonconsumers of RM or PM in the reference group (nonconsumer reference) compared with studies where the reference included nonconsumers and consumers of low levels of RM or PM (mixed-consumer reference). We then identified and added additional studies with a nonconsumer reference group via a narrative review (see Supplemental Materials 1 for inclusion and exclusion criteria).

**FIGURE 1:**
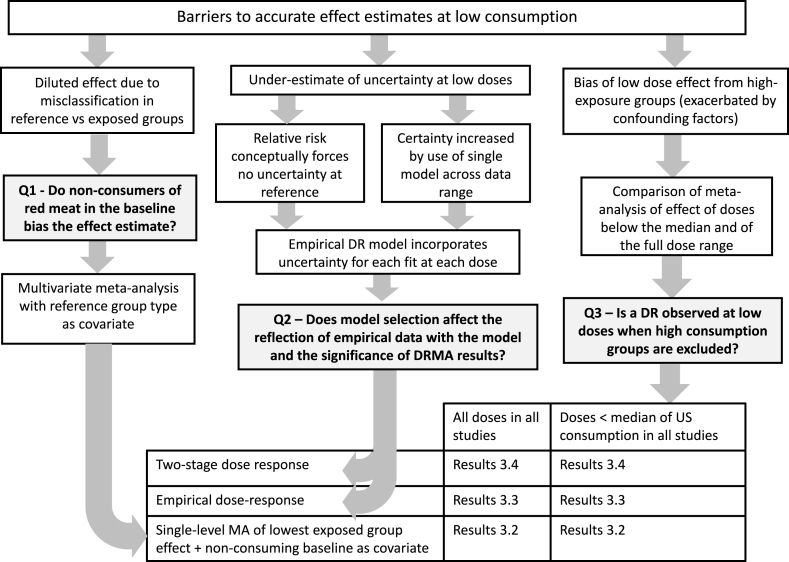
Approach to evaluating 3 hypotheses on barriers for accurate estimation of effects at low doses using observational DR. Darker boxes indicate questions to be addressed, connected by arrows to the different models and data used to address those questions (bottom table). Sections that present the results of each test are listed in the table. DR, dose response; MA, Meta-Analysis.

We included for analysis (Figure 1) studies in any population that included CRC incidence and mortality outcomes, and clearly characterized the consumption of fresh RM or PM (that is, studies reporting a combination of RM and PM were excluded from analysis).

The presence of very low consumers in the reference groups could potentially mask the association between CRC and RM or PM consumption, as has been observed in studies of environmental exposures [[33], [34], [35]]. Thus, we carried out a meta-regression to estimate this association at low consumption levels (that is, the lowest consumption groups in the included studies), and tested whether this association was statistically different between nonconsumer and mixed reference studies.

We then fitted DRMA using multiple methods, described in detail below. The goals of this meta-analysis were as follows: *1*) to evaluate a possible DR association between RM or PM and CRC at low consumption levels, *2*) to evaluate a possible DR association between RM or PM and CRC at all consumptions considered in the included studies, and *3*) to assess the impact of high consumers on the DR estimation at low consumption values. Further, we *4*) evaluate the impact that model selection has on DR estimates above by comparing assorted DR method results, and 5) we assess whether the presence of RM or PM consumers in the study’s reference group affects the DR association. As shown in Figure 1, objectives 1-3 address question 3, objective 4 addresses question 2, and objective 5 addresses question 1.

Data were extracted in MS Excel version 2310 [36]. All analyses were performed using the R statistical language version 4.2.2 [37]. Multivariate meta-regression models were implemented using the *metafor* package 4.4–0 [38], and DRMA models were implemented in *dosresmeta* 2.0.1 [39]. We followed Meta-analysis Of Observational Studies in Epidemiology (MOOSE) guidelines for reporting the methodology and results.

### Literature search

The systematic review by the GBD on RM and PM as dietary risk factors, which are carried out and reported according to PRISMA and Guidelines for Accurate and Transparent Health Estimates Reporting (GATHER) standards, formed the basis of the literature used in this analysis. To maximize comparability with the results of the GBD DR as reported in the risk factor analysis from 2019 [29], we collected all studies that were cited by the GBD project systematic reviews on the topic of RM or PM as of 2020. The initial list therefore included studies resulting from a search of the GHDx [31] for the terms “red meat,” “processed meat,” “red,” “processed,” and “meat.” We further added any additional studies listed as references for past risk factor assessment publications [29,[40], [41], [42], [43], [44]], either directly cited for RRs for RM and PM impacts or those contained references to protein sources, RM, and/or PM in the title or keywords. As all studies were initially reviewed, rated for risk of bias, and selected for inclusion during the GBD systematic review, only an evaluation of the specificity of consumption (that is, separation of unprocessed RM and PM) was performed.

To identify further studies with a nonconsumer reference group, we additionally performed a narrative review using the MEDLINE, Scopus, Cochrane Library, PubMed, and Google Scholar databases in February 2021. Search terms included “vegetarianism” or “vegetarian” or “meatless” and “colorectal” or “colon” or “rectal” and “cancer.” We initially reviewed the studies for relevancy to the exposure (that is, consumption of unprocessed RM or PM specifically) and the health outcomes of interest, retaining only cohort studies that provided RR estimates of colorectal, rectal, or colon cancer for categories of meat consumption amounts.

The resulting list of studies were reviewed independently in duplicate and selected based on the inclusion criteria (Supplemental Materials 1). Source studies cited in reviews or meta-analyses were reviewed individually to avoid repeatedly including studies or cohorts. The key characteristics for inclusion in the meta-analysis were specificity of consumption (separate characterization of risks from fresh RM and from PM consumption) and of outcome (incidence or mortality of CRCs); quantitative consumption reporting for the consumption groups either as or convertible to g/d; and type of study—cross-sectional studies were omitted from this analysis. If >1 study examined the same cohort of participants and met all inclusion criteria, we selected the study that covered the longest time period, or, duration being equal, was the most recent. This literature review and meta-analysis are consistent with MOOSE standards for reporting (Supplemental Material 2).

### Data processing

Data extraction from the selected studies was performed in duplicate and cross-checked. We recorded or converted from available data, if not directly reported, consumption of RM /PM in g/d. For studies that recorded consumption as servings per day, week, or month, we applied the study-specific serving size if reported. In the absence of a reported serving size, we sought other publications or the food-frequency questionnaire used for the study’s cohort to identify a serving size. If no cohort-specific source could be identified, we applied a serving size from another study in the same country, or the same population. This last substitution was required for cohorts from 2 studies: the Melbourne Collaborative cohort [45], for which portion sizes from the Australian National Nutrition and Physical Activity Survey were used [46], and the Women’s Health Study [47], which reported using the same questionnaire as the Nurses’ Health Study (NHS) [48], and thus, the NHS serving size was used. For studies that reported consumptions as grams per caloric intake, the average caloric intake—per consumption group if reported by the study, or overall for the cohort if per group was not reported—was used to convert the consumption to g/d.

For 6 of the 24 included studies where the person-years consumption per group was not available, we calculated it by multiplying the number of participants per group by the mean follow-up duration reported by the article. In the 12 studies where only the total number of participants was reported, we divided the total number of participants by the number of consumption groups to estimate the number of participants per group.

### Meta-analyses

#### CRC association with low consumption of RM and PM, and the effect of low-level consumers in the reference group

To test for an association between low consumption levels of RM or PM and CRC, we performed a random-effects (RE) meta-analysis of only study groups with the lowest consumption above the reference group. For example, if a study population was divided into quintiles and thus had a reference group as the lowest quintile and 4 consumption groups, we used only the RR estimate for the second quintile. To adjust for varying consumption levels between studies, we used the median consumption amount per group as a covariate. Further, we used an indicator variable to evaluate whether the association was different between studies with mixed-consumer reference groups compared with those with strictly nonconsumers in the reference, as established with 2-sided *t*-test of slopes.

#### Identification of lower and higher consumption groups

Consumption groups were classified as “lower consumption” if their median consumption was less than the median consumption of the United States population (21 g/d and 56 g/d for PM and RM, respectively) as measured by NHANES [49]. Study groups with consumptions at or above those values were considered “higher consumption.” Although meat consumption varies across nations, 43% of the person-years included were from United States cohorts (Table 1) [27,28,45,47,[50], [51], [52], [53], [54], [55], [56], [57], [58], [59], [60], [61], [62], [63], [64], [65], [66], [67], [68], [69]], and the person-year weighted median consumption of the United States cohorts and non-United States cohorts in this analysis were similar (40 g and 42 g/d RM, respectively, and 22 and 28 g/d PM, respectively). Because the data do not indicate substantial dissimilarity, we applied the United States-based limits to all cohorts.

**TABLE 1:** Cohorts included in analysis and their characteristics, including average duration of follow-up (in y), the total number of participants and cases, person-years contributed by the participants, the food assessed (PM = processed meat, RM = red meat), outcome, and reference group (either mixture of nonconsumers and consumers or nonconsumers only)

Lead author and year	Cohort	Duration	Total N	Cases N	Person-years	Food assessed	Outcome	Reference group	Consumption range (RM) (g/d)	Consumption range (PM) (g/d)	Cited by GBD project	Outcome type	Country	Outlying consumers omitted	PM definition
Chao et al. [50] (2005)	CPS II (males)	9	69,664	665	626,976	PM/RM	CRC Incidence	Mixture	14–143	0–21	Yes	HR	United States	Yes (based on energy)	Uncertain, probably red PM only
Chao et al. [50] (2005)	CPS II (females)	9	78,946	532	710,514	PM/RM	CRC Incidence	Mixture	6–102	1–41	Yes	HR	United States	Yes (based on energy)	Uncertain, probably red PM only
Cross et al. [51] (2010)	NIH-AARP	7	300,948	2719	2,106,636	PM	CRC Incidence	Mixture	NA	2–44	Yes	HR	United States	Yes (based on energy)	Mixture of red and white PM
Diallo et al. [52] (2018)	NutriNet-Santé	4.1	61,476	120	252,052	PM/RM	CRC Incidence	Mixture	3–94	3–39	Yes	HR	France	No	“Mostly” red PM
Egeberg et al. [53] (2013)	Danish Diet, Cancer, and Health	13.4	25,832	664	346,149	PM/RM	Colon Cancer Incidence	Mixture	48–126	8–54	Yes	HR	Denmark	No	“Mostly” red PM
English et al. [45] (2004)	Melbourne Collaborative	9	37,112	451	334,008	PM/RM	CRC Incidence	Mixture	30–157	5–33	Yes	HR	Australia	Yes (based on energy)	Uncertain, probably mixture of red and white PM
Flood et al. [54] (2003)	Breast Cancer Detection Demonstration	11	45,496	528	500,456	PM	CRC Incidence	Mixture	NA	8–64	Yes	HR	United States	Yes (based on energy and excluded 65 reported meat frequency of >9×/d)	Uncertain, probably mixture of red and white PM
Gilsing et al. [55] (2015)	The Netherlands Cohort	20.3	10,210	437	207,263	PM/RM	CRC Incidence	Nonconsumers	0–133	2–25	Yes	HR	The Netherlands	No	Mixture of red and white PM
Knuppel et al. [56] (2020)	UK Biobank	6.9	474,996	3228	3,277,472	PM/RM	CRC Incidence	Mixture	6–55	4–29	No	HR	United Kingdom	No	Mixture of red and white PM
Larsson et al. [57] (2005)	Swedish Mammography	13.9	61,443	735	854,058	PM/RM	CRC Incidence	Mixture	18–67	6–41	Yes	HR	Sweden	Yes (based on energy)	Uncertain, probably red PM only
Lin et al. [47] (2004)	Women’s Health Study	8.7	37,547	202	326,659	PM	CRC Incidence	Nonconsumers	NA	0–23	Yes	HR	United States	Yes (based on energy)	Uncertain, probably red PM only
Mehta et al. [58] (2020)	Sister Study	8.7	48,704	216	423,725	PM/RM	CRC Incidence	Mixture	7–54	4–28	No	HR	United States	Yes (based on energy)	Mixture of red and white PM
Mejborn et al. [59] (2020)	Danish National Survey on Diet and Physical Activity	10.9	6282	640	68,474	PM/RM	CRC Incidence	Mixture	20–151	9–99	No	HR	Denmark	No	Mixture of red and white PM
Oba et al. [27] (2006)	Japanese males	7	13,894	111	97,258	PM/RM	CRC Incidence	Mixture	19–57	4–20	Yes	HR	Japan	No	Uncertain, probably red PM only
Oba et al. [27] (2006)	Japanese females	7	16,327	102	114,289	PM/RM	CRC Incidence	Mixture	10–42	3–16	Yes	HR	Japan	No	Uncertain, probably red PM only
Ollberding et al. [60] (2012)	Multiethnic cohort	13.6	165,717	3404	2,253,751	PM/RM	CRC Incidence	Mixture	9–79	3–41	Yes	HR	United States	Yes (based on energy, fat, protein)	Red PM only
Parr et al. [61] (2013)	Norwegian Women and Cancer	11.1	84,538	674	938,372	PM/RM	CRC Incidence	Mixture	3–42	9–71	Yes	HR	Norway	Yes (based on energy)	Uncertain, probably red PM only
Pietinen et al. [28] (1999)	Alpha-Tocopherol, Beta-Carotene	8	27,111	185	216,888	PM	CRC Incidence	Mixture	NA	26–122	Yes	HR	Finland	No	Red PM only
Sato et al. [62] (2006)	Miyagi Cohort	11	41,836	358	460,196	PM	CRC Incidence	Mixture	NA	0–16	Yes	RR	Japan	Yes (based on energy)	Red PM only
Sellers et al. [63] (1998)	Iowa Women’s Health (with family history)	10	4239	61	42,390	PM/RM	CRC Incidence	Mixture	21–122	1–16	Yes	HR	United States	Yes (based on energy)	Uncertain, probably red PM only
Sellers et al. [63] (1998)	Iowa Women’s Health (with no family history)	10	22,698	180	226,980	PM/RM	CRC Incidence	Mixture	21–122	1–16	Yes	HR	United States	Yes (based on energy)	Uncertain, probably red PM only
Takachi et al. [64] (2011)	Japan Public Health Center men	9.4	38,462	481	361,543	PM	Colon Cancer Incidence	Mixture	NA	0.4–15	Yes	HR	Japan	Yes (based on weight)	Mixture of red and white PM
Takachi et al. [64] (2011)	Japan Public Health Center females	9.4	42,196	307	396,642	PM	Colon Cancer Incidence	Mixture	NA	0.4–14	Yes	HR	Japan	Yes (based on weight)	Mixture of red and white PM
Tiemersma et al. [65] (2002)	Dutch Monitoring Project on CVD	8.5	639	102	5,432	RM	CRC Incidence	Mixture	24–109	NA	Yes	OR	The Netherlands	Yes (based on energy)	
Vulcan et al. [66] (2017)	Malmö Diet and Cancer	18	27,931	923	502,758	PM	CRC Incidence	Mixture	NA	19–92	Yes	HR	Sweden	No	Red PM only
Wada et al. [67] (2017)	Takayama males	16	13,957	429	223,312	PM/RM	CRC Incidence	Mixture	19–64	4–23	Yes	RR	Japan	No	Uncertain, probably red PM only
Wada et al. [67] (2017)	Takayama females	16	16,374	343	261,984	PM/RM	CRC Incidence	Mixture	12–49	3–19	Yes	RR	Japan	No	Uncertain, probably red PM only
Ward et al. [68] (2016)	EPIC	10.5	519,978	3789	5,459,769	PM/RM	CRC Mortality	Mixture	9–91	5–66	Yes	HR	10 EU countries	Yes (based on energy)	Uncertain, probably red PM only
Wei et al. [69] (2004)	HPFS	6	46,632	668	279,792	PM/RM	Colon Cancer Incidence	Nonconsumers	0–120	0–64	Yes	HR	United States	Yes (based on energy)	Uncertain, probably mixture of red and white PM
Wei et al. [69] (2004)	NHS	6	87,733	467	526,398	PM/RM	Colon Cancer Incidence	Nonconsumers	0–120	0–64	Yes	HR	United States	Yes (based on energy)	Uncertain, probably mixture of red and white PM

The consumption range in the study (based on the median of each group) and whether the study is cited in the GBD project is also included. PM definitions that are described as “mostly” red PM describe those studies that did not explicitly omit processed white meat from the PM definition, but which indicated that white PM consumption was low or nonexistent in the cohort. PM definitions described as “uncertain” were not specific enough in their food description to definitively conclude the PM definition did not include processed white meats.

Abbreviations: CPS, Cancer Prevention Study; CRC, colorectal cancer; CVD, Cardiovascular Disease; EPIC, European Prospective Investigation into Cancer and Nutrition; GBD, Global Burden of Disease; HPFS, Health Professionals Follow-Up Study; HR, Hazard Ratio; NA, not applicable; NHS, Nurses’ Health Study; NIH-AARP, National Institutes of Health - American Association of Retired Persons.

#### DRMA methods to test significance of DR association at lower consumption levels and the impact of model selection on data representation

We used pointwise meta-regressions described by Crippa et al. [70] to create an empirical summary of the evidence of a dose-response (empirical DR) based on the data from studies included. In short, this method allows for the fit of a unique DR function per study, and the predictions from each fit are combined using standard random effects (RE) meta-analysis methods. This provides an accurate reflection of the data from each study, in contrast to conventional DR methods that impose a single mathematical relationship between consumption and health outcome.

We fitted an empirical DR to the lower consumption groups from all the studies, and contrasted it against the fit using all consumption groups. The choice of model was made from 46 possible candidate models fitted (see Supplemental Table 1 for full description of each model) to each study in the data set. We selected the best-fitting model based on a combination of appropriate degrees of freedom, visual inspection to ascertain the model predictions fall within reported confidence intervals (CIs), and lowest Akaike Information Criteria (AIC). For example, studies that had only 2 consumption arms could not be fitted with a cubic spline, and studies with only 1 consumption arm could only be fitted with intercept-only or linear models. For studies where the regression parameters were nonsignificant, an intercept-only model was chosen to reflect the lack of a DR in the study, avoiding variance shrinkage in the RR at the reference consumption amount. Within the consumption range covered by each study, we used the best model fit to predict the RR at each consumption in g/d and combined these predictions via an RE meta-analysis per consumption amount. For comparison, we constructed a conventional 2-stage DR meta-analysis model for both lower consumption groups and all consumption groups of RM and PM using restricted cubic spline (RCS) using 3 knots at the 5th, 65th, and 95th percentiles of the consumption.

The GBD [29] provided RR predictions at 25 g/d intervals. We linearly extrapolated their estimates at 1 g/d intervals between the reported RR to provide a more continuous estimate for comparison against the other models.

### Imputation of highest group consumption

The classic method for imputation is to add the distance between the median and range in the second highest consumption group to the highest group minimum. This approach assumes that the consumption distribution is not highly skewed. As consumption is often skewed [71], this imputation might underestimate consumption, resulting in an overestimation of the slope of the DRMA, which will affect the RRs in the lower consumption range (see Supplemental Figure 1 for an example using NHANES data) [22,72]. For studies where the median and maximum of the highest consumption categories were not reported (e.g., greater than *x* g/d), we extrapolated the median consumption by 2 different methods. In the “classic” version, we applied the default method used in nutritional DRMA [3,73,74] where the median of the highest group was estimated as the minimum consumption of that group plus the distance between that minimum and the median of the second highest consumption group. In the “probabilistic” method, we used interval-censored maximum likelihood estimation [75] to fit Weibull and Gamma distributions to the g/d amounts reported for all other consumption categories for each study, and the fit with the lowest AIC was selected. The values corresponding to the median of the upper consumption group were then estimated from the study-specific distribution based on the appropriate percentile. For example, the midpoint of the uppermost quintile of a distribution corresponds to the 90th percentile. This provided a consistent and robust extrapolation of the highest consumptions based on the entirety of the consumption data, in contrast to using the interval in just one consumption group under the default method. An illustrative comparison between methods is provided in Supplemental Figure 1 and the resulting differences in consumption and DR estimation are demonstrated in Supplemental Figure 2.

### Comparison of DRMA models

For each model, we predicted the mean and 95% CI RR in 1 g/d intervals within the range of consumptions in the studies analyzed. RRs between models were considered not significantly different if the CIs overlapped. RRs were considered statistically significant if the CIs did not overlap with the null (RR< >1).

## Results

### Data summary

**FIGURE 2:**
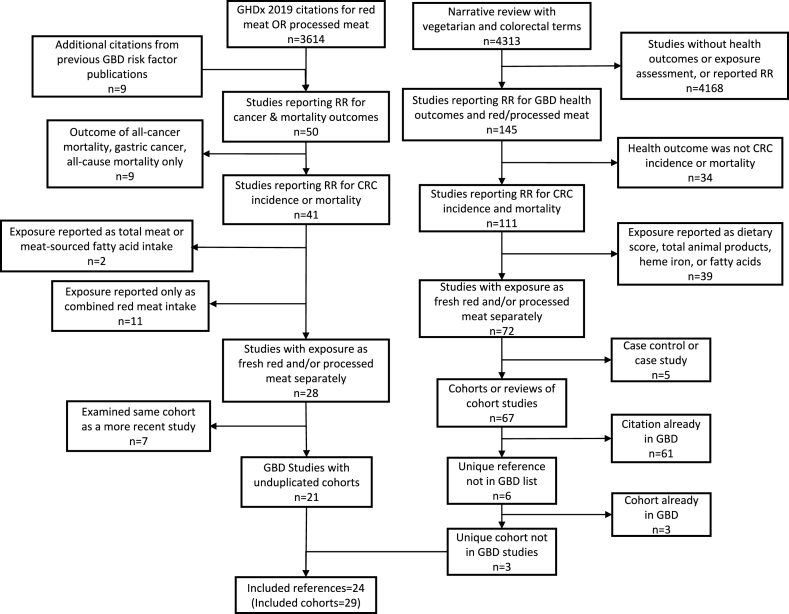
Inclusion and exclusion of studies from the Global Burden of Disease (GBD) project citation lists and the narrative review to arrive at the data set used in these analyses.

From the initial 3164 studies in GHDx, only 41 remained after exclusion of studies without reported RRs associating CRC/mortality and RM or PM consumption. Nine studies from the meta-analysis used in previous iterations of the GBD risk factor analyses for CRC and RM or PM were also considered to reflect the complete evidence base of the GBD project, since former decisions to include a risk factor influence present-day inclusion [29]. These 50 studies were reviewed by the two authors to establish whether the study met inclusion criteria for the meta-analysis for CRC (Supplemental Table 2). Of these studies, 9 [[76], [77], [78], [79], [80], [81], [82], [83], [84]] were excluded because the outcome was not specific to colon or rectal cancer (that is, total mortality, total cancer incidence or mortality, or gastrointestinal tract cancer), and 2 [85,86] for reporting consumption only as total meat intake or total fatty acid intake. Eleven studies [[87], [88], [89], [90], [91], [92], [93], [94], [95], [96], [97]] were excluded for reporting consumption only as combined RM intake, rather than stratified as processed and fresh RM. Seven studies [[98], [99], [100], [101], [102], [103], [104]] were excluded because they examined duplicate cohorts. From the remaining 21 studies for inclusion in the analysis, 7 reported a nonconsumer reference group either for RM or PM (Figure 2).

The narrative review for dietary studies with nonconsumer reference groups for PM and RM returned 4313 initial results. Of these, based on the abstract review, 67 were cohort studies that evaluated consumption per study group of RM or PM and examined CRC as the outcome. Of these, 61 studies were already included in the reference list from the GHDx, leaving 6 references not included in the GBD-based review. Three of these examined the same cohort already included by ≥1 GBD reference and were excluded [[105], [106], [107]], and so we included 3 additional studies, all of which were published in 2020 [56,58,59].

The combined list of references from which data was applied in the analysis included 24 publications (Figure 2) [27,28,45,47,[50], [51], [52], [53], [54], [55], [56], [57], [58], [59], [60], [61], [62], [63], [64], [65], [66], [67], [68], [69]]. We extracted 29 separate cohorts (Table 1) [27,28,45,47,[50], [51], [52], [53], [54], [55], [56], [57], [58], [59], [60], [61], [62], [63], [64], [65], [66], [67], [68], [69]] from these studies, including those that reported stratified results by sex [27,50, 67,69] or by family history [63] as separate cohorts.

In total the data set for PM contained data from 29 cohorts for 2,662,065 individuals, who contributed 23,522,676 person-years and 20,818 cases of colon or rectal cancer. All outcomes in the PM data set were reported as RRs (2 publications) or hazard ratios (21 publications), with median group consumption ranging from 0 to 122 g/d (median 14 g/d). The data set for RM contained 1,887,743 individuals from 23 cohorts who contributed 15,365 cases of colon or rectal cancer over 17,259,839 person-years, and the range of median consumption was 0–157 g/d (median 38.5). Only 1 nested case-control study [65], reported odds ratios, whereas the others reported RRs (1 publication) or hazard ratios (17 publications). Omission of the single case-control, which included 639 individuals, did not alter the analysis results. To remain consistent with the GBD data set, and given the similarity of the research question of the case-control study [108], we retained this study in the analysis.

Only Ward et al. [68] used an outcome of mortality from CRC rather than diagnosis [68]. Although this study contributes ∼5.5 million person-years to both the PM and RM data sets, removal of this study shows no significant impact on the results (Supplemental Tables 3 and 4). To remain consistent with the GBD data set, we retained this study in the analysis.

In the lower consumption version of the PM data, 28 cohorts were used ([28] was omitted as the lowest consumption in this study was 26 g/d). Those groups that qualified as lower consumers totaled 1,502,129 people, who contributed 13,190,459 person-years. The median amount of PM per day in these lower consumption groups ranged from 0 to 19.8 g/d, with a median of 7.5. In the lower consumers of RM, 23 cohorts were still applicable, and 1,486,516 people contributed 13,374,839 person-years. The median consumption of this lower consumer database for RM ranged from 0 to 55.1, with a median of 24.4 g/d. In 7 of the studies, only the reference group was low enough to qualify as lower consumption, and therefore, these studies were omitted from the lower consumption DRMA but were used in the DRMA of all consumption ranges [45,53,59,61,63,65,66].

### Impact of nonconsumer compared with lowest consumption group as reference

**FIGURE 3:**
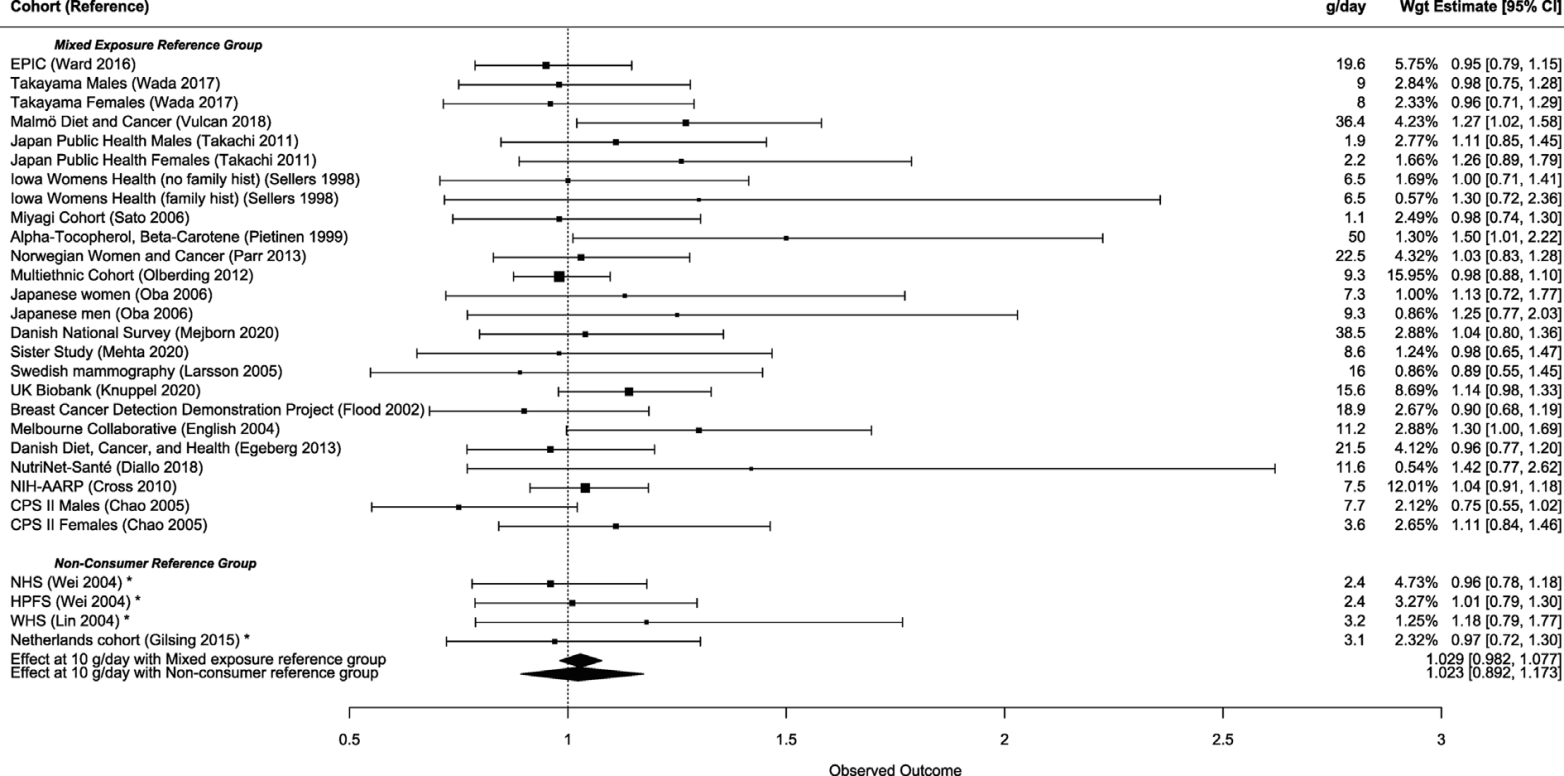
Multivariate meta-analysis of lowest-dose group for PM with RR at 10 g/d and mixed consumption or no-consumption in the reference group. PM, processed meat.

**FIGURE 4:**
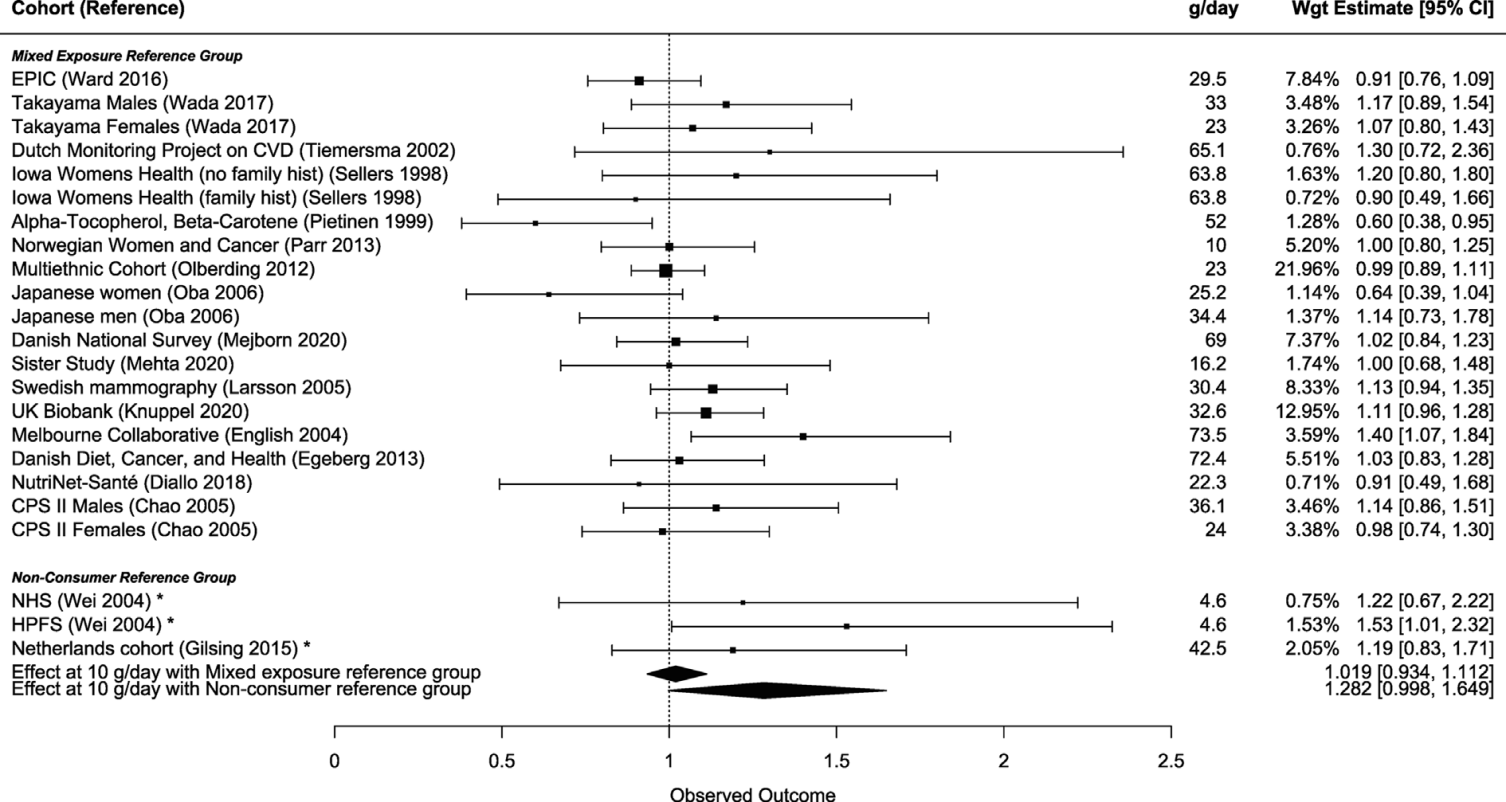
Multivariate meta-analysis of lowest consumption group for RM with RR at 10 g/d and mixed consumption or no-consumption in the reference group. RM, unprocessed red meat.

Those in the lowest consumption groups above the reference eat 12.5 (1.87–42.95) g/d of PM and 37.0 (4.6–72.9) g/d of RM. Using an RE meta-regression, we found no association between CRC and the lowest-level consumption of PM (*P* = 0.16) and RM (*P* = 0.30) across studies, and this association was not influenced by the presence of a nonconsumer reference in PM (*P* = 0.94) or RM (*P* = 0.06) (Supplemental Table 5). Although we found no DR at these consumption levels, we predicted the RR at 10 g/d to adjust for differences in consumption between studies, and we found no statistically significant difference in the predicted RR between nonconsumer 1.02 (0.892–1.173) and mixed-consumer reference groups 1.03 (0.982–1.077) for PM studies (Figure 3). Likewise, the RRs were not different at 10 g/d of RM when the reference had nonconsumers 1.28 (0.998–1.165) compared with mixed-consumers 1.02 (0.934–1.112) (Figure 4).

### Empirical DR fits

Although 46 candidate models were fitted to each study, only a few model types consistently provided the best fit (Table 2) [27,28,45,47,[50], [51], [52], [53], [54], [55], [56], [57], [58], [59], [60], [61], [62], [63], [64], [65], [66], [67], [68], [69]]. Examples of the potential fits resulting from each model relative to the data from the study are shown in the Supplemental Figures 3–6). In 4 studies of PM and in 2 studies of RM, as no RR was significant, the best fitting model was an intercept-only model (Table 2 and Supplemental Figures 3–6) [27,28,45,47,[50], [51], [52], [53], [54], [55], [56], [57], [58], [59], [60], [61], [62], [63], [64], [65], [66], [67], [68], [69]]. The intercept-only model was more commonly used in the lower consumption range models, especially because a few studies in both PM [27,54,56,57,68] and RM [27,28,50,55] data sets (Table 2) [27,28,45,47,[50], [51], [52], [53], [54], [55], [56], [57], [58], [59], [60], [61], [62], [63], [64], [65], [66], [67], [68], [69]] had only 1 consumption group other than the reference group below the median consumption cut-off, so that insufficient degrees of freedom were present to fit most regressions. In 61 of 72 models that were not intercept-only and where the data had sufficient degrees of freedom to support an RCS model, RCS provided a better fit than linear, exponential, quadratic, or fractional polynomial regressions (Table 2) [27,28,45,47,[50], [51], [52], [53], [54], [55], [56], [57], [58], [59], [60], [61], [62], [63], [64], [65], [66], [67], [68], [69]]. No consistent pattern was present even for those studies where RCS provided the best fit. For example, some studies showed a positive trend with meat consumption, whereas others showed a negative trend, or highly nonlinear trends (Supplemental Figures 3–6)

**TABLE 2:** Model fit characteristics for each individual cohort as used in the empirical dose-response model. Best-fitting models for lower consumption and all consumption data are listed under “Model applied columns”. The significance of the linear dose-response or trend analysis as fit by the original study authors is listed under “DR reported by study”, and the lowest consumption group with significant effect above the reference group is also reported under “Lowest significant arm”

Lead author and year	Cohort	Meat type	Consumption median (g/d)	Model applied in empirical DR method (lower consumption)	Model applied in empirical DR method (all consumers)	DR reported by study	Lowest significant arm
Chao et al. [50] (2005)	CPS II (males)	PM	14	RCS	RCS	Linear (*P* = 0.03)	None
Chao et al. [50] (2005)	CPS II (females)	PM	6	RCS	RCS	Linear (*P* = 0.02)	None
Cross et al. [51] (2010)	NIH-AARP	PM	18	RCS	RCS	Linear (*P* = 0.017)	12.1 g/1000 kcal
Diallo et al. [52] (2018)	NutriNet-Santé	PM	19	Intercept only	Intercept only	Linear (*P* = 0.6)	None
Egeberg et al. [53] (2013)	Danish Diet, Cancer, and Health	PM	27	NA	*β*_0_+*β*_1_∗log(*x*)^3^+*β*_2_∗log(*x*)^2^	Linear (*P* = 0.53)	None
English et al. [45] (2004)	Melbourne Collaborative	PM	13	RCS	RCS	Linear (*P* = 0.2)	4+ servings/wk
Flood et al. [54] (2003)	Breast Cancer Detection Demonstration	PM	27	Intercept only	RCS	Linear (*P* = 0.35)	None
Gilsing et al. [55] (2015)	The Netherlands Cohort	PM	12	*β*_0_+*β*_1_∗*x*^−2^+*β*_2_∗log(*x*) ^−2^	RCS	Linear (*P* = 0.05)	None
Knuppel et al. [56] (2020)	UK Biobank	PM	16	Intercept only	RCS	Linear (*P* = 0.0023)	21.3 g/d
Larsson et al. [57] (2005)	Swedish Mammography	PM	25	Intercept only	RCS	Linear (*P* = 0.23)	None
Lin et al. [47] (2004)	Women’s Health Study	PM	8	RCS	RCS	Linear (*P* = 0.25)	None
Mehta et al. [58] (2020)	Sister Study	PM	11	Intercept only	RCS	Linear (*P* = 0.02)	28.1 g/d
Mejborn et al. [59] (2020)	Danish National Survey on Diet and Physical Activity	PM	35	NA	RCS	Linear (*P* = 0.34)	None
Oba et al. [27] (2006)	Japanese males	PM	9	Intercept only	RCS	Linear (*P*<0.01)	20.3 g/d
Oba et al. [27] (2006)	Japanese females	PM	7	RCS	RCS	Linear (*P* = 0.62)	None
Ollberding et al. [60] (2012)	Multiethnic Cohort	PM	16	RCS	RCS	Linear (*P* = 0.156)	None
Parr et al. [61] (2013)	Norwegian Women and Cancer	PM	30	NA	RCS	Linear (*P* = 0.02)	>60 g/d
Pietinen et al. [28] (1999)	Alpha-Tocopherol, Beta-Carotene	PM	62	NA	RCS	Linear (*P* = 0.78)	None
Sato et al. [62] (2006)	Miyagi Cohort	PM	2.5	Intercept only	Intercept only	Linear (*P* = 0.38)	None
Sellers et al. [63] (1998)	Iowa Women’s Health (with family history)	PM	12	RCS	RCS	Linear (*P* = 0.9)	None
Sellers et al. [63] (1998)	Iowa Women’s Health (with no family history)	PM	12	Intercept only	Intercept only	Linear (*P* = 1.0)	None
Takachi et al. [64] (2011)	Japan Public Health Center males	PM	7	RCS	RCS	Linear (*P* = 0.1)	None
Takachi et al. [64] (2011)	Japan Public Health Center women	PM	7	RCS	RCS	Linear (*P* = 0.64)	None
Vulcan et al. [66] (2017)	Malmö Diet and Cancer	PM	40	NA	Intercept only	Linear (*P* = 0.012)	0.025 g/MJ
Wada et al. [67] (2017)	Takayama males	PM	13.3	Intercept only	*β*_0_+*β*_1_∗x^3^+*β*_2_∗log(*x*)^3^	Linear (*P* = 0.23)	None
Wada et al. [67] (2017)	Takayama females	PM	11	RCS	*β*_0_+*β*_1_∗x^3^+*β*_2_∗log(*x*)^3^	Linear (*P* = 0.95)	None
Ward et al. [68] (2016)	EPIC	PM	27	Intercept only	RCS	Linear (*P* = 0.46)	None
Wei et al. [69] (2004)	HPFS	PM	12	*β*_0_+*β*_1_∗x^−2^+*β*_2_∗log(*x*) ^−2^	RCS	Linear (*P* = 0.37)	None
Wei et al. [69] (2004)	NHS	PM	8	*β*_0_+*β*_1_∗x^−2^+*β*_2_∗log(*x*) ^−2^	RCS	Linear (*P* = 0.02)	None
Chao et al. [50] (2005)	CPS II (males)	RM	68	Intercept only	RCS	Linear (*P* = 0.03)	None
Chao et al. [50] (2005)	CPS II (females)	RM	39	Intercept only	RCS	Linear (*P* = 0.48)	None
Diallo et al. [52] (2018)	NutriNet-Santé	RM	43	Intercept only	Intercept only	Linear (*P* = 0.5)	None
Egeberg et al. [53] (2013)	Danish Diet, Cancer, and Health	RM	100	NA	RCS	Linear (*P* = 0.2)	None
English et al. [45] (2004)	Melbourne Collaborative	RM	86	NA	RCS	Linear (*P* = 0.01)	3.0–4.4 servings/wk
Gilsing et al. [55] (2015)	The Netherlands Cohort	RM	71	Intercept only	Intercept only	Linear (*P* = 0.12)	None
Knuppel et al. [56] (2020)	UK Biobank	RM	38	RCS	RCS	Linear (*P*<0.0085)	55.1 g/d
Larsson et al. [57] (2005)]	Swedish Mammography	RM	60	RCS	RCS	Linear (*P* = 0.32)	None
Mehta et al. [58] (2020)	Sister Study	RM	30	RCS	Intercept only	Linear (*P* = 0.76)	None
Mejborn et al. [59] (2020)	Danish National Survey on Diet and Physical Activity	RM	65	NA	RCS	Linear (*P* = 0.85)	None
Oba et al. [27] (2006)	Japanese males	RM	33	Intercept only	RCS	Linear (*P* = 0.86)	None
Oba et al. [27] (2006)	Japanese females	RM	22	Intercept only	RCS	Linear (*P* = 0.20)	None
Ollberding et al. [60] (2012)	Multiethnic Cohort	RM	40	RCS	RCS	Linear (*P* = 0.064)	None
Parr et al. [61] (2013)	Norwegian Women and Cancer	RM	20	Intercept only	Intercept only	Linear (*P* = 0.45)	None
Pietinen et al. [28] (1999)	Alpha-Tocopherol, Beta-Carotene	RM	60	NA	RCS	Linear (*P* = 0.74)	None
Sellers et al. [63] (1998)	Iowa Women’s Health (with family history)	RM	56	NA	RCS	Linear (*P* = 0.3)	None
Sellers et al. [63] (1998)	Iowa Women’s Health (with no family history)	RM	56	NA	RCS	Linear (*P* = 0.6)	None
Tiemersma et al. [65] (2002	Dutch Monitoring Project on CVD	RM	44	NA	RCS	Linear (*P* = 0.1)	None
Wada et al. [67] (2017)	Takayama males	RM	41	RCS	RCS	Linear (*P* = 0.009)	64 g/d
Wada et al. [67] (2017)	Takayama females	RM	30	RCS	RCS	Linear (*P* = 0.98)	None
Ward et al. [68] (2016)	EPIC	RM	40	RCS	RCS	Linear (*P* = 0.053)	None
Wei et al. [69] (2004)	HPFS	RM	26	β_0_+β_1_∗log(×)^–0.5^	β_0_+β_1_∗log(×)^–0.5^	Linear (*P* = 0.70)	<0-3 servings/ month
Wei et al. [69] (2004)	NHS	RM	31	Intercept only	Intercept only	Linear (*P* = 0.44)	None

Abbreviations: CPS, Cancer Prevention Study; CVD, Cardiovascular Disease; DR, dose response; EPIC, European Prospective Investigation into Cancer and Nutrition; HPFS, Health Professionals Follow-Up Study; NHS, Nurses' Health Study; NIH-AARP, National Institutes of Health - American Association of Retired Persons; PM, processed meat; RCS, restricted cubic spline; RM, unprocessed red meat.

### Comparison of lower consumption DR with all consumption

**FIGURE 5:**
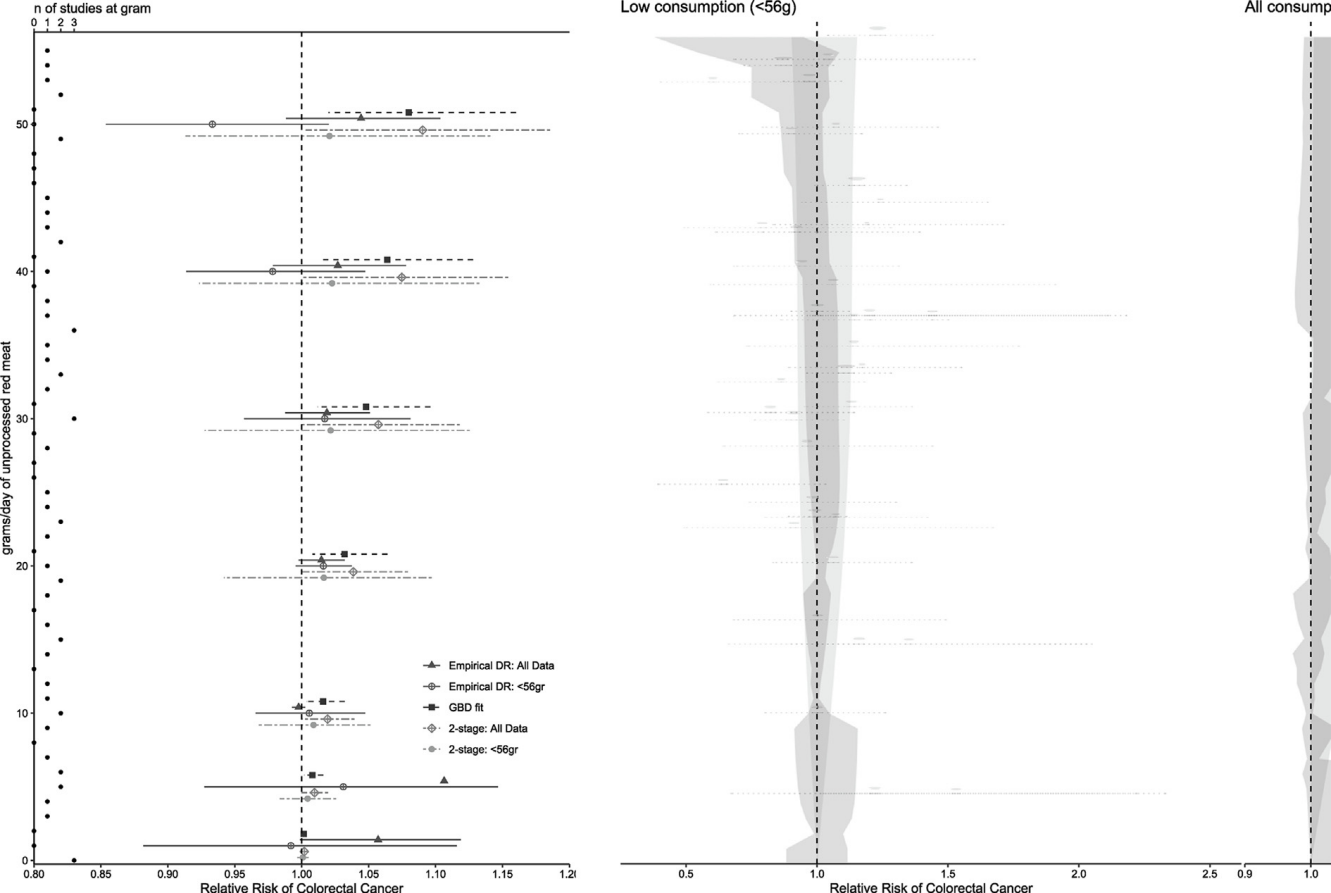
Predictions from empirical DR model and 2-stage RCS model fit to lower consumption data (<56 g/d) for unprocessed red meat and colorectal cancer compared with the GBD fit and with the underlying data (per-exposure group relative risks reported in each source study) (middle) with predictions for specific consumption amounts highlighted (left side), compared against the 2-stage RCS model and empirical DR fit to all consumption levels (right). DR, dose response; GBD; Global Burden of Disease; RCS, restricted cubic spline.

**FIGURE 6:**
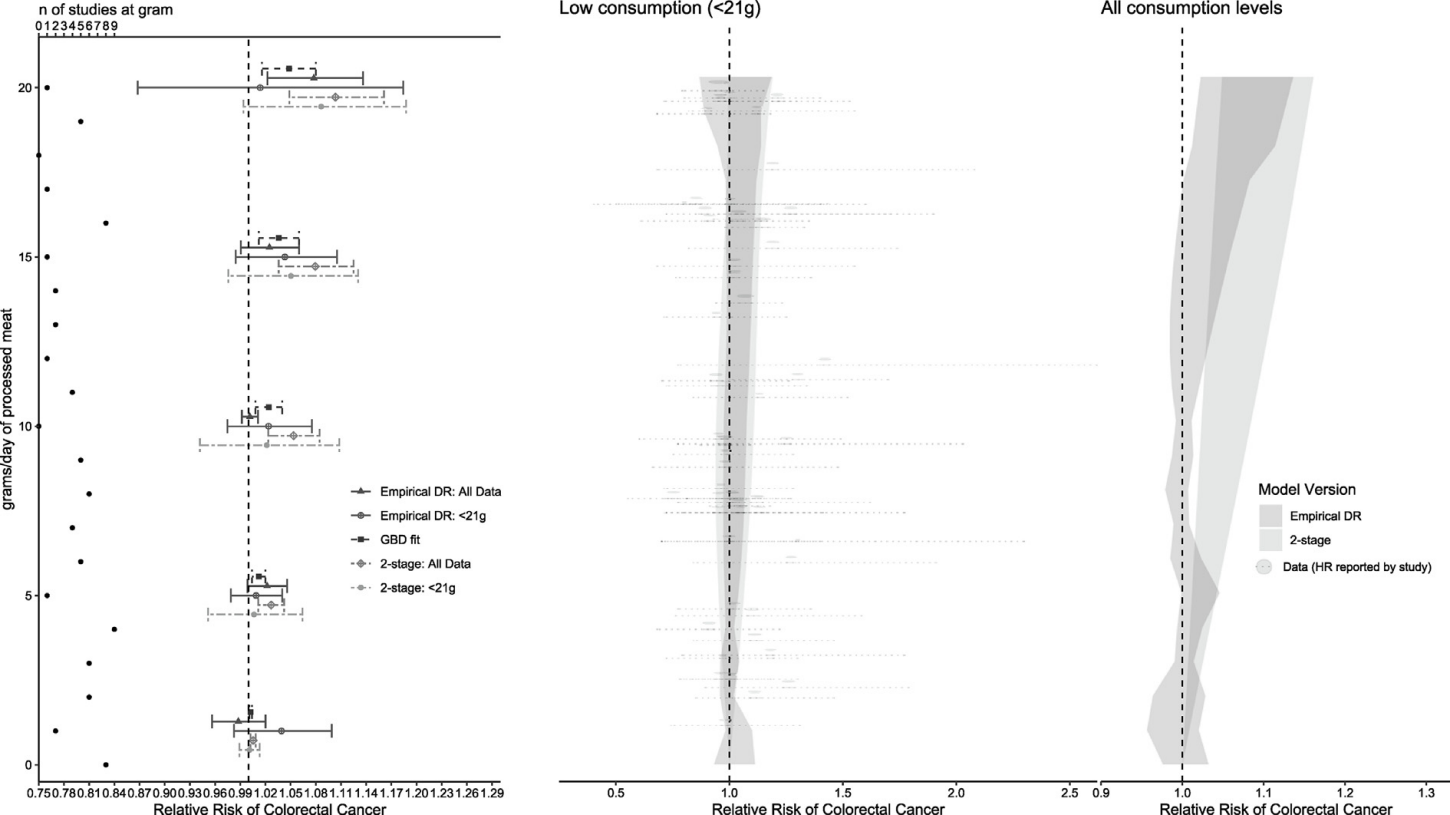
Predictions from empirical DR model and 2-stage RCS model fit to lower consumption data (<21 g/d) for processed meat and colorectacl cancer compared with the GBD fit and with the underlying data (per-exposure group relative risks reported in each source study) (middle) with predictions for specific consumption amounts highlighted (left side), compared against the 2-stage RCS model and empirical DR fit to all consumption levels (right). DR, dose response; GBD; Global Burden of Disease; RCS, restricted cubic spline.

Neither the empirical DR nor 2-stage models fit to the lower consumption data sets showed significant effects of RM or of PM on CRC (FIGURE 5, FIGURE 6) within the lower consumption range (20 g or less for PM and 56 g or less for RM). The estimated RR at an amount of 10 g/d consumption of PM and RM as estimated by all 4 of our models and by the linear extrapolation between the effects reported by the GBD is listed in Table 3.

**TABLE 3:** Prediction of RR at 10 g/d and highest consumption with no effect relative to zero consumption as predicted by 2-stage RCS DRMA and empirical DR models for both unprocessed red meat and processed meat, for lower consumers only, and for all consumption levels in range of the component studies, with the linearly extrapolated fits from GBD 2019 for comparison

Model version	Consumption range	Model	Cohorts (*n*)	Total (*n*)	Total cases	RR at 10 g/d	Highest consumption in data without effect (g)
Processed meat models							
Empirical LC	0–20 g/d	Empirical DR	24	2,367,463	10,611	1.021 (0.97–1.07)	>20
2-stage LC	0–20 g/d	2-stage RCS	24	2,367,463	10,611	1.016 (0.94–1.10)	>20
Empirical all	0–122 g/d (all data)	Empirical DR	29	2,539,157	20,818	1.000 (0.99–1.01)	5
2-stage all	0–122 g/d (all data)	2-stage RCS	29	2,539,157	20,818	1.052 (1.02–1.08)	0
GBD	0–100 g/d	GBD (linear extrap)	unknown	unknown	unknown	1.024 (1.01–1.04)	0
Unprocessed red meat models							
Empirical LC	0–56 g/d	Empirical DR	17	1,827,921	10,746	0.999 (0.99–1.01)	>56
2-stage LC	0–56 g/d	2-stage	17	1,827,921	10,746	1.007 (0.97–1.05)	>56
Empirical all	0–157 g/d (all data)	Empirical DR	23	1,924,723	15,365	0.998 (0.99−1.01)	30
2-stage all	0–157 g/d (all data)	2-stage	23	1,924,723	15,365	1.019 (0.99–1.04)	34
GBD	0–200 g/d	GBD (linear extrap)	Unknown	Unknown	Unknown	1.016 (1.00–1.03)	0

Abbreviations: DR, dose response; DRMA, dose-response meta-analysis; GBD; Global Burden of Disease; LC, lower consumers; RCS, restricted cubic spline.

### Comparison of empirical DR against 2-stage RCS model

The 2-stage RCS models fit to the lower consumptions (<21 g/d PM and <56 g/d RM) yielded similar results to the empirical DR (FIGURE 5, FIGURE 6 and Table 3), in that neither DRMA method identified significant associations when fitted using only data below these low-consumption thresholds for either PM or RM. However, these model types differ in the low consumer data in the greater smoothness of the CIs of the 2-stage model and in their ability to reflect the underlying uncertainty in the source study data. Compared with the empirical DR that combines a variety of models including intercept-only, the 2-stage model shows much less statistical uncertainty at the lowest ranges of consumption (Supplemental Figures 7 and 8), for models of RM and PM, and therefore does not reflect the uncertainty of the low-consumption arms of individual studies within the data sets well.

When fitted using the full range of consumptions in each study, the empirical DR model for RM identified a transient significant effect above 30 g/d, but at amounts between 36 and 84 g/d, the RR became again nonsignificant. Using the 2-stage model, a significant effect of RM on CRC incidence began at 34 g/d and was observed ≤147 g/d. The empirical DR model appears more sensitive both to nonmonotonicity and to changes in the number of studies that provide data at any given consumption amount. Both models show potential for nonmonotonic DR relationships with a nonzero threshold between RM and CRC. In both cases, the use of all consumption amounts introduced a significant effect at a consumption below 56 g/d, which was not observed when using lower consumption data (Table 3 and Figure 5, Supplemental Tables 6–9), reflecting a change in the DR relationship above the lower consumers that can impact the entire DR curve, especially in 2-stage models. The GBD DRMA model showed a significant effect of consumption beginning at 0 g/d, given the extrapolation between the zero consumption and the lowest reported RR (25 g/d), consistent with the monotonicity assumed by the researchers.

In the case of PM, a similar pattern to RM was found in that no significant association between PM and CRC was observed below 21 g/d using either the 2-stage or the empirical DR when using only data below that threshold. Again, when compared with the empirical DR, the 2-stage model shows an artificially narrow uncertainty at the lowest ranges of consumption (Supplemental Figures 6 and 7). When using all consumption ranges from the same source studies, the 2-stage model found a significant association at any consumption, but the empirical model’s association began at 5 g/d. This significant RR persisted only until 7 g/d, however, and then remained nonsignificant ≤17 g/d (Figure 6 and Table 3). The CIs of the empirical DR, without the additional smoothing found in the 2-stage models, were less consistently above or below the null in the PM model (Supplemental Tables 6 and 7 and Figure 6) compared with the 2-stage and GBD models. The GBD DRMA increased linearly from zero and was significant at all consumptions, as in the case of RM.

## Discussion

Our analysis explored the association between consumption of RM and PM and CRC, and how the choice of data and models can affect these estimates, particularly at low consumption levels. We concluded that no significant association between RM and PM and CRC could be observed at consumptions below median consumption in the United States when data from higher consumers is excluded. Empirical DRMA methods better reflected the nonmonotonicity and uncertainty of the DRMA models, and demonstrated that relationships between risk of CRC and consumption of RM or PM may differ among higher consumers, or that residual confounding may play a biasing role in estimates of this DR that use methods that extrapolate from high to low consumption levels.

A unique aspect of our analysis is the use of a fully empirical DR model for each study that was included in the DRMA. For example, if a study reported consumption between 10 and 30 g/d of RM, with the empirical DR fit, only model predictions from that study within that range would be used in the DRMA. This is in contrast with the assumption made with 2-stage models, which assume an a priori functional form of the DR (e.g., linear), and because of this assumption allow for extrapolation outside of the data observed per study. Our empirical approach resulted in nonsmooth, nonmonotonic relationships that apply fewer assumptions to the DR, in contrast with some estimates reported in literature. For example, the World Cancer Research Fund (WCRF) assumed a linearly increasing risk of CRC over the full range of consumption levels of RM or PM, which resulted in an estimated 6% increased risk at 50 g/d of RM [109]. In contrast, using the empirical DRMA, we found no association [1.04 (0.99–1.10)] between CRC and RM at 50 g/d. In addition, the empirical DR estimates do not show a consistent pattern across the lower consumers - the consumption levels most often observed - even in populations where per-capita consumption is relatively high. For example, two-thirds of United States consumers of RM eat 63 g/d or less [49]. At 63 g/d, the 2-stage RCS model estimates an RR of 1.11 (1.01–1.22), but the empirical DR method does not identify a consistently elevated risk until 84 g/d (three-fourths of United States consumers fall below this level). Although the GBD 2019 used a more empirical approach than a purely linear assumption by applying an spline-based method, it did assume monotonicity in their DRMA for RM and PM [29]. This assumption was applied to create a consistent curve that presents a more biologically plausible relationship, according to the manuscript's authors [110]. However, we found evidence of nonmonotonicity here—the association increased and became significant in some ranges followed by a decline back to nonsignificance, and was drawn away from the null when using higher ranges of consumption, indicating a change in the relationship at higher consumption—which explains why our DRMA findings differ despite using equivalent data.

Adding to the evidence against monotonicity, we found that higher consumption amounts (above 56 g/d RM or 21 g/d PM) do bias the association between CRC and consumption below these amounts of RM or PM. For RM, the RR at 50 g/d when using an empirical fit with lower consumption [0.93 (0.85–1.02)] or when estimated with all consumption levels [1.04 (0.99–1.10)], were both nonsignificant. This finding is consistent with the majority of the studies, where no significant association with CRC was found at most RM consumption levels (Table 2). For PM, the RR at 20 g/d was not statistically significant 1.01 (0.87–1.18) when using only lower consumption evidence, consistent with the majority of individual study results in lower consumers (Table 2), and significantly >1 at a consumption level of 20 g/d [1.07 (1.02–1.12)] when all consumption data was used. This bias was magnified with 2-stage fits, where adding higher consumption data resulted in a statistically significant association between CRC and consumptions over between 30 and 34 g/d of RM, whereas we found no association when using only lower consumption levels with the 2-stage DR or with the empirical DR method. Similarly, using lower consumption (below 56 g/d RM or 21 g/d PM) evidence, we found no association using the 2-stage model between CRC and PM consumption under 20 g/d, whereas using all consumption levels resulted in a statistically significant association with PM consumption of as little as 1 g/d.

A DR model based on data originating from studies with randomized experimental designs minimizing the potential for confounding would support using the full range of consumption in the data set even in the case of these types of nonmonotonic relationships. However, in observational studies, the possibility of consumption misclassification and residual confounding can rarely be fully discounted and may be greater or smaller depending on whether all studies control for the same covariates, and the strength of the methods for controlling for these covariates. In such cases, monotonicity that accurately reflects a different biological relationship above a certain consumption level (that is, a threshold in the DR) and a spurious relationship because of residual confounding may be difficult to distinguish, and therefore, a DRMA using a large range of data should be used with caution.

Differences between the empirical and 2-stage DR were also observed in the amount of uncertainty captured by the CIs, especially at the lowest consumption levels, as evident from the model predictions for the individual studies using a 2-stage DR (Supplemental Figures 7 and 8) and resulting overall association with CRC (FIGURE 5, FIGURE 6). The low number of studies that contribute data at consumption below 10 g/d of RM (only 3 studies had a nonreference group with <10 g/d consumption) is better reflected by this empirical DR method because it assumes no extrapolation outside of study data. The empirical approach avoids underestimating the uncertainty at consumption levels with little underlying data, which can be a pitfall of methods that require the same model be fit to each study, or methods that fit a single model to all available data. Underestimation of uncertainty because of the fitting of a nonempirical model, especially at low consumption, can also lead to considerable underestimates of risk-free consumption levels when the DR relationship is used to derive values such as the theoretical minimum risk exposure level (TMREL). Empirical DR fitting methods can more accurately estimate uncertainty across the DR curve compared with other methods that extrapolate beyond individual studies to reduce uncertainty. Our findings using an empirical DR to reflect the uncertainty of the underlying data do not support a TMREL of zero, that is, the assumption that there is no safe RM or PM consumption level with regard to CRC. Such TMRELs are used by groups such as the GBD to estimate burden associated with risk factors such as RM consumption, and their values can therefore have a considerable effect on dietary guidelines.

Intuitively, if there is a monotonic dose-dependent health effect from an exposure, studies with reference groups that only include nonexposed individuals (non-RM or PM consumers) should show a stronger RR than those where the reference group includes some exposure/consumption. When we evaluated this hypothesis, we did not find such an effect when separating studies with a nonconsumer reference. This finding could also lend support to the idea that residual confounding inordinately influences the estimates at high consumption ranges. It should be noted that we only found 3 cohorts with nonconsumers of RM as the reference so our findings might have been underpowered to detect such differences. Thus, future cohort publications should identify and separate nonconsumers for their reference groups when reporting RRs.

A limitation of the empirical method is the potential loss of continuity in the DR curve, particularly where there are dose ranges with few or no data points, but this issue is a reflection of the underlying data, which may exhibit noncontinuous and nonmonotonic trends that can be smoothed over by more widely used modeling methods such as the 2-stage model. In the case of nutritional epidemiological studies, especially for lifetime consumption habits and noncommunicable disease outcomes such as CRC, DR methods that are suitable for experimental data and that use a wide range of consumptions to reduce overall model uncertainty may not be appropriate because of residual confounding at high consumption and imprecision in consumption measurements.

Our study is limited by issues arising from common practices in nutritional cohort studies. The use of categorized consumptions, especially when represented by a median or mean amount in grams or servings per day, overstates the certainty of consumption amounts, particularly over a lifetime. Our analysis likely underestimates consumption uncertainty in the DR models for this reason. Monte Carlo simulation methods can be used to propagate the consumption uncertainty through the DR relationship, increasing the models’ prediction intervals. We did not carry out this estimation to be more comparable to other reported DR methods, and because many studies do not report consumption uncertainty per group. The use of open-ended consumption amounts in the highest consumer groups, another common practice that we partially address using a probabilistic method, also may underestimate within-group variability, given the skewedness of consumption distributions in most populations.

The importance of data from low-consumption ranges is highlighted in this study, especially when the goal is to understand risks of populations whose consumption is largely or entirely below levels where elevated RR are observed in individual studies. Even in countries with relatively high RM consumption [111], most RM consumers fall below the 100 g/d amount reported to summarize linear DR models. Nonlinearity and nonmonotonicity of DR at population-relevant levels of consumption should also be considered in the development of dietary recommendations, because both aspects of DR models are highly relevant to what constitutes a risk-free level of consumption. Our findings show that after analyzing 22,402,195 total person-years and using empirical models that fit each study independently, lower consumption of RM or PM is not associated with an increased risk of CRC. We also show that other reports might have overestimated this association because of modeling assumptions and/or the influence of inclusion of higher consumption amounts into their estimates. Finally, we show that a TMREL of 0 consumption of RM and PM is inconsistent with the evidence presented in this study.

## Acknowledgments

We thank Dr Macon Overcast for his assistance in reviewing articles and extracting study data, and Dr. Solenne Costard for her valuable editorial feedback during the drafting of this manuscript.

## Author contributions

The authors’ contributions were as follows – JGP, FJZ: designed research; JGP: reviewed studies and extracted data for analysis; FJZ, JGP: analyzed data and performed statistical analysis and wrote the article; JGP: has primary responsibility for final content; and all authors: read and approved the final manuscript.

## Conflict of interest

JGP, FJZ work for EpiX Analytics. EpiX Analytics reports consulting and research engagements for MatPrat.

## Funding

MatPrat Norway provided partial funding for the analysis supporting this manuscript. MatPrat had no role in the collection, analysis, or reporting of this data, no role in preparation of this manuscript, and no role in the decision for publication.

## Data availability

Data described in the manuscript, code book, and analytic code will be made available upon request.
