# Supplementary material for: Observational Dose-Response Meta-Analysis Methods May Bias Risk Estimates at Low Consumption Levels: The Case of Meat and Colorectal Cancer
Source: Adv Nutr. 2024 Mar 21;15(5):100214. doi: 10.1016/j.advnut.2024.100214 (PMC11061242; doi:10.1016/j.advnut.2024.100214)
Supplement: Multimedia Component.1 [file mmc1.docx]

Supplemental Materials

Supplemental Material 1: Inclusion Criteria for all studies

- 1. Included studies must be cohort studies, randomized control studies, or nested case-control studies.
  2. Included studies must be peer-reviewed.
  3. Included studies must report a measure of relative risk (Risk Ratio, Hazard Ratio, or Odds Ratio) for outcomes of colorectal cancer incidence or colorectal cancer mortality, or alternatively colon cancer incidence or colon cancer mortality (as colon cancer represents most of the cases of the combination of colon and rectal cancers).
  4. Included studies must report exposure as unprocessed red meat and/or processed meat separately, not as combined red meat or total meat.
  5. Included studies must report exposure in a measure that is able to be converted to grams/day for each exposure group.
  6. Included studies must not report data from a cohort which is already included in the analysis via a more recent study of the same person-years.

Supplemental Material 2: MOOSE Checklist:

Supplemental Material 3: Tables and Figures

Supplemental Table 1: Candidate models fit to each study individually for selection of the best fit and subsequent assembly into the empirical DR.

| Model description | Formula |
| --- | --- |
| Fractional Polynomials where p = all combinations of {−2, −1, −0.5, 0, 0.5, 1, 2, 3} | Functions with powers (p1, p2) include the formulations β_0_+β_1_X^p1^ + β_2_X^p2^ and  β_1_X^p1^ + β_2_Z^p1^ log(X) if p2 = p1 |
| 1/(x+1) | y=β_0_+β_1_X^-1^ |
| 1/sqrt(x+1) | y=β_0_+β_1_X^-0.5^ |
| sqrt(x) | y=β_0_+β_1_X^0.5^ |
| 1/(x+1)^3^ | y=β_0_+β_1_X^-3^ |
| X^2^ | y=β_0_+β_1_X^2^ |
| 1/(x+1)^2^ | y=β_0_+β_1_X^-2^ |
| Intercept-only | y=β_0_ |
| linear | β_0_+β_1_Z |
| Restricted cubic spline | 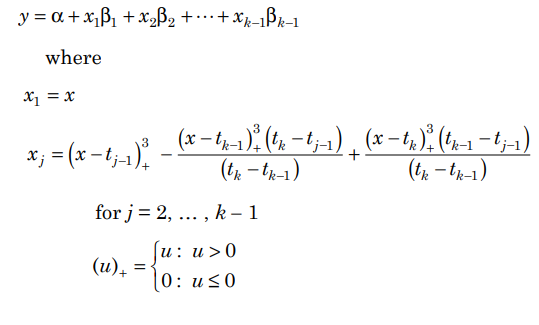 |


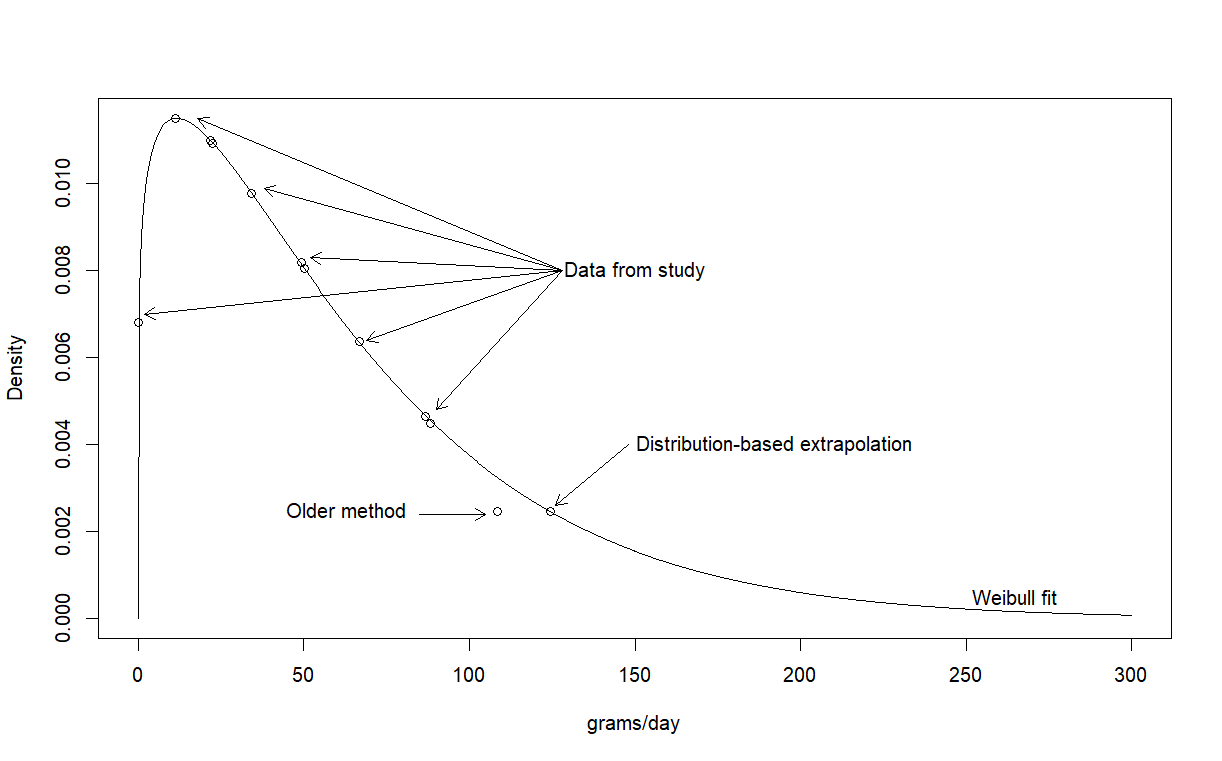


Supplemental Figure 1: Example comparison of two methods to impute median consumption (g/day) in the highest consumption group, using data on unprocessed red meat consumption from the NHANES study. The “classic method” adds the distance between the median and range in the second highest consumption group to the highest group minimum. The “probabilistic method” is the result of an interval-censored maximum likelihood estimation fit to a Weibull distribution, and then calculating the distribution’s percentile to impute the median consumption in the highest consumption group.


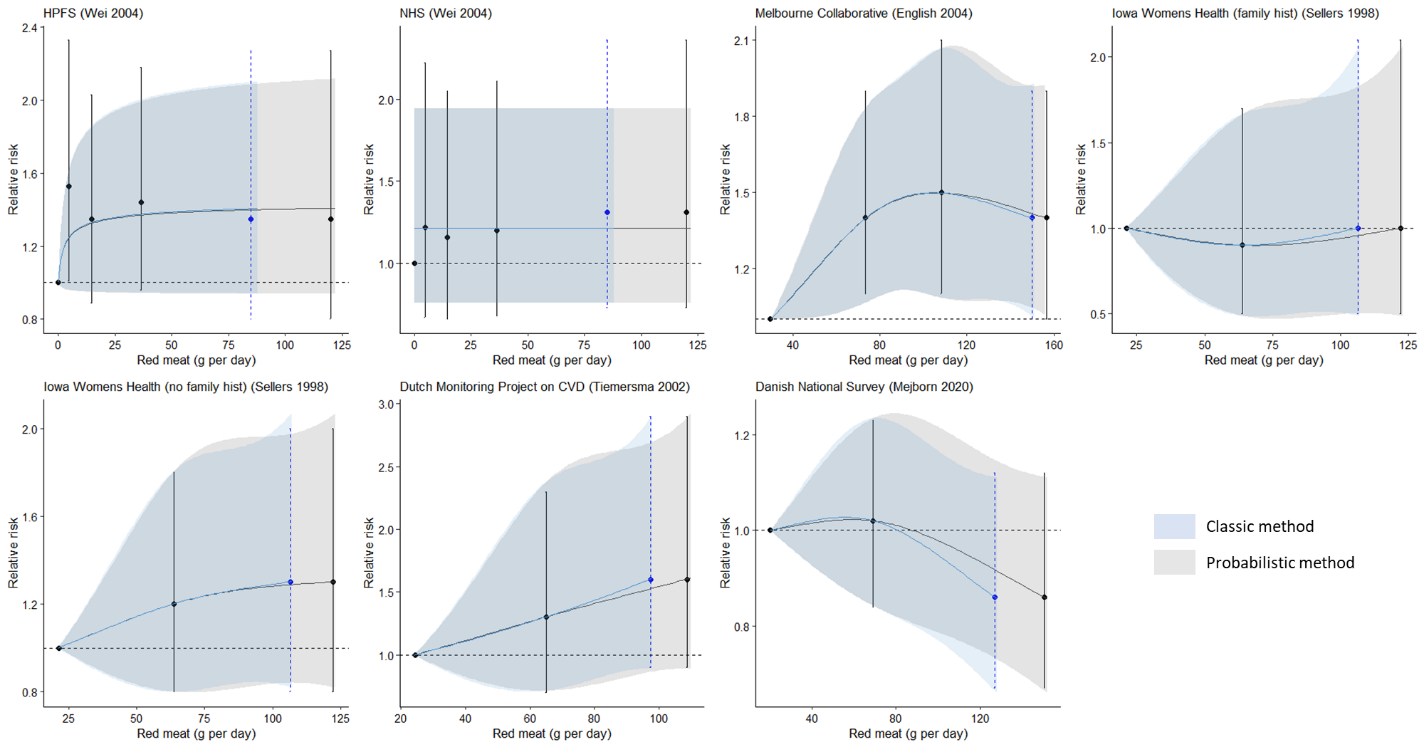


Supplemental Figure 2: Difference in consumption amounts and dose-response model fits to seven example RM studies based on two approaches to extrapolating the median dose of the upper consumption group, the classic method shown in blue and the probabilistic method shown in grey.

Supplemental Table 2: Included and Excluded studies following elimination of studies which did not include outcomes of cancer mortality in both the narrative search and review of the Global Burden of Disease Project input sources.

| Study | Included /Excluded | Reason for exclusion |
| --- | --- | --- |
| Aleksandrova K, Pischon T, Jenab M, Bueno-de-Mesquita HB, Fedirko V. et al. Combined impact of healthy lifestyle factors on colorectal cancer: a large European cohort study. BMC Med. 2014 Oct 10;12:168. doi: 10.1186/s12916-014-0168-4. PMID: 25319089; PMCID: PMC4192278. | Excluded | relative risks are only reported for healthy lifestyle score |
| Alshahrani SM, Fraser GE, Sabaté J, Knutsen R, Shavlik D, Mashchak A, Lloren JI, Orlich MJ. Red and Processed Meat and Mortality in a Low Meat Intake Population. Nutrients. 2019; 11(3). | Excluded | Outcome is all cancer mortality |
| Appleby PN, Crowe FL, Bradbury KE, Travis RC, Key TJ. Mortality in vegetarians and comparable nonvegetarians in the United Kingdom. *Am J Clin Nutr*. 2016;103(1):218-230. doi:10.3945/ajcn.115.119461 | Excluded | exposure is to total meat |
| Balder HF, Vogel J, Jansen MCJF, Weijenberg MP, van den Brandt PA, Westenbrink S, van der Meer R, Goldbohm RA. Heme and chlorophyll intake and risk of colorectal cancer in the Netherlands cohort study. Cancer Epidemiol Biomark Prev. 2006; 15(4): 717–25. | Excluded | Cohort already included in a more recent study (Gilsing 2015) |
| Beeson, W.L., Mills, P.K., Phillips, R.L., Andress, M. and Fraser, G.E. (1989), Chronic disease among seventh-day adventists, a low-risk group. Rationale, methodology, and description of the population. Cancer, 64: 570-581. https://doi.org/10.1002/1097-0142 | Excluded | exposure is to total meat, cohort already included via later study |
| Bellavia A, Stilling F, Wolk A. High red meat intake and all-cause cardiovascular and cancer mortality: is the risk modified by fruit and vegetable intake?. Am J Clin Nutr. 2016; 104(4): 1137-1143. | Excluded | Outcome is all cancer mortality |
| Bostick RM, Potter JD, Kushi LH, Sellers TA, Steinmetz KA, McKenzie DR, Gapstur SM, Folsom AR. Sugar, meat, and fat intake, and non-dietary risk factors for colon cancer incidence in Iowa women (United States). Cancer Causes Control. 1994; 5(1): 38-52. | Excluded | cohort already included in a more recent study (Sellers 1998) |
| Brink M, Weijenberg MP, de Goeij AF, Roemen GM, Lentjes MH, de Bruïne AP, Goldbohm RA, van den Brandt PA. Meat consumption and K-ras mutations in sporadic colon and rectal cancer in The Netherlands Cohort Study. Br J Cancer. 2005; 92(7): 1310-20. | Excluded | exposure is total meat |
| Butler LM, Wang R, Koh WP, Yu MC. Prospective study of dietary patterns and colorectal cancer among Singapore Chinese. Br J Cancer. 2008 Nov 4;99(9):1511-6. doi: 10.1038/sj.bjc.6604678. Epub 2008 Sep 23. PMID: 18813309; PMCID: PMC2579684. | Excluded | exposure is dietary pattern (vegetarian or not), no meat percentile characterization |
| Chao A, Thun MJ, Connell CJ, McCullough ML, Jacobs EJ, Flanders WD, Rodriguez C, Sinha R, Calle EE. Meat consumption and risk of colorectal cancer. JAMA. 2005; 293(2): 172-82. | Included |  |
| Chen JJ, Stampfer MMJ, Hough HHL, et al. A prospective study of N-acetyltransferase genotype, red meat intake, and risk of colorectal cancer. Cancer Res 1998;58:3307-11. | Excluded | exposure is to combined red meat |
| Cross AJ, Leitzmann MF, Gail MH, Hollenbeck AR, Schatzkin A, Sinha R. A prospective study of red and processed meat intake in relation to cancer risk. PLoS Med. 2007; 4(12): e325. | Excluded | Cohort already included in a more recent study (Cross 2010) |
| Cross AJ, Ferrucci LM, Risch A, Graubard BI, Ward MH, Park Y, Hollenbeck AR, Schatzkin A, Sinha R. A large prospective study of meat consumption and colorectal cancer risk: an investigation of potential mechanisms underlying this association. Cancer Res. 2010; 70(6): 2406–14. | Included (PM only) |  |
| Diallo A, Deschasaux M, Latino-Martel P, Hercberg S, Galan P, Fassier P, Allès B, Guéraud F, Pierre FH, Touvier M. Red and processed meat intake and cancer risk: Results from the prospective NutriNet-Santé cohort study. Int J Cancer. 2018; 142(2): 230-237. | Included |  |
| Diergaarde B, Braam H, van Muijen GNP, Ligtenberg MJ, Kok FJ, Kampman E. Dietary factors and microsatellite instability in sporadic colon carcinomas. Cancer Epidemiol Biomarkers Prev. 2003;12:1130–6 | Excluded | combined red meat as exposure, also case control study |
| Dixon LB, Balder HF, Virtanen MJ, Rashidkhani B, Männistö S, Krogh V, van Den Brandt PA, Hartman AM, Pietinen P, Tan F, Virtamo J, Wolk A, Goldbohm RA. Dietary patterns associated with colon and rectal cancer: results from the Dietary Patterns and Cancer (DIETSCAN) Project. Am J Clin Nutr. 2004 Oct;80(4):1003-11. doi: 10.1093/ajcn/80.4.1003. PMID: 15447912. | Excluded | relative risks are only reported for dietary scores |
| Dominguez LJ, Bes-Rastrollo M, Basterra-Gortari FJ, Gea A, Barbagallo M, Martínez-González MA. Should we recommend reductions in saturated fat intake or in red/processed meat consumption? The SUN prospective cohort study. Clin Nutr. 2018; 37(4): 1389-1398. | Excluded | Outcome is all cancer mortality |
| Egeberg R, Olsen A, Christensen J, Halkjær J, Jakobsen MU, Overvad K, Tjønneland A. Associations between red meat and risks for colon and rectal cancer depend on the type of red meat consumed. J Nutr. 2013; 143(4): 464-72. | Included |  |
| English DR, MacInnis RJ, Hodge AM, Hopper JL, Haydon AM, Giles GG. Red meat, chicken, and fish consumption and risk of colorectal cancer. Cancer Epidemiol Biomark Prev. 2004; 13(9): 1509–14. | Included |  |
| Etemadi A, Sinha R, Ward MH, Graubard BI, Inoue-Choi M, Dawsey SM, Abnet CC. Mortality from different causes associated with meat, heme iron, nitrates, and nitrites in the NIH-AARP Diet and Health Study: population based cohort study. BMJ. 2017; 357: j1957. | Excluded | Outcome is total cancer and not CRC |
| Evans, Richard C. et al. Diet and colorectal cancer: An investigation of the lectin/galactose hypothesis Gastroenterology, Volume 122, Issue 7, 1784 - 1792 | Excluded | exposure is to combined red meat |
| Farvid MS, Malekshah AF, Pourshams A, Poustchi H, Sepanlou SG, Sharafkhah M, et al. Dietary Protein Sources and All-Cause and Cause-Specific Mortality: The Golestan Cohort Study in Iran. Am J Prev Med. 2017; 52(2): 237-248. | Excluded | Outcome is GI cancer or all mortality |
| Fernandez, E., Negri, E., La Vecchia C., Franceschi, S. Diet Diversity and Colorectal Cancer, Preventive Medicine, Volume 31, Issue 1, 2000, Pages 11-14, ISSN 0091-7435, https://doi.org/10.1006/pmed.2000.0667. | Excluded | exposure is to total meat, characterized as meat diversity. |
| Ferrucci LM, Cross AJ, Graubard BI, Brinton LA, McCarty CA, Ziegler RG, Ma X, Mayne ST, Sinha R. Intake of meat, meat mutagens, and iron and the risk of breast cancer in the Prostate, Lung, Colorectal, and Ovarian Cancer Screening Trial. Br J Cancer 2009; 101: 178–84. | Excluded | exposure is to combined red meat |
| Flood A, Velie EM, Sinha R, Chaterjee N, Lacey JV, Schairer C, Schatzkin A. Meat, fat, and their subtypes as risk factors for colorectal cancer in a prospective cohort of women. Am J Epidemiol. 2003; 158(1): 59–68. | Included (PM only) |  |
| Fortes C, Forastiere F, Farchi S, Rapiti E, Pastori G, Perucci CA. Diet and overall survival in a cohort of very elderly people. Epidemiology. 2000; 11(4): 440-5. | Excluded | exposure is combined red meat |
| Franceschi, S., Favero, A., La Vecchia, C., Negri, E., Conti, E., Montella, M., Giacosa, A., Nanni, O. and Decarli, A., 1997. Food groups and risk of colorectal cancer in Italy. International Journal of Cancer, 72(1), pp.56-61. | Excluded | total meat as exposure |
| Fraser GE. Associations between diet and cancer, ischemic heart disease, and all-cause mortality in non-Hispanic white California Seventh-day Adventists. Am J Clin Nutr 1999;70:532S-8S. | Excluded | exposure is combined red meat |
| Frentzel-Beyme R, Chang-Claude J. Vegetarian diets and colon cancer: the German experience. Am J Clin Nutr. 1994 May;59(5 Suppl):1143S-1152S. doi: 10.1093/ajcn/59.5.1143S. PMID: 8172115. | Excluded | exposure is vegetarian/non vegetarian, i.e., total meat consumption |
| Gilsing AM, Schouten LJ, Goldbohm RA, Dagnelie PC, van den Brandt PA, Weijenberg MP. Vegetarianism, low meat consumption and the risk of colorectal cancer in a population based cohort study. Sci Rep. 2015; 5: 13484. | Included |  |
| Giovannucci E, Rimm EB, Stampfer MJ, et al. Intake of fat, meat, and fiber in relation to risk of colon cancer in men. Cancer Res 1994;54:2390-7. | Excluded | a more recent study uses the same cohort (HPFS) |
| Godos J, Bella F, Sciacca S, Galvano F, Grosso G. Vegetarianism and breast, colorectal and prostate cancer risk: an overview and meta-analysis of cohort studies. J Hum Nutr Diet. 2017 Jun;30(3):349-359. doi: 10.1111/jhn.12426. Epub 2016 Oct 6. PMID: 27709695. | Excluded | Review study with no new sources beyond existing list |
| Goldbohm RA, van den Brandt PA, van ‘t Veer P, et al. A prospective cohort study on the relation between meat consumption and the risk of colon cancer. Cancer Res 1994;54:718-23. | Excluded | Cohort already included in a more recent study (Gilsing 2015) |
| Gunter, M.J., Probst-Hensch, N.M., Cortessis, V.K., Kulldorff, M., Haile, R.W. and Sinha, R., 2005. Meat intake, cooking-related mutagens and risk of colorectal adenoma in a sigmoidoscopy-based case-control study. Carcinogenesis, 26(3), pp.637-642. | Excluded | exposure is to combined red meat |
| Hsing AW, McLaughlin JK, Chow WH, Schuman LM, Co Chien HT, Gridley G, Bjelke E, Wacholder S, Blot WJ. Risk factors for colorectal cancer in a prospective study among U.S. white men. Int J Cancer. 1998; 77(4): 549-53. | Excluded | exposure is combined red meat |
| Hutter CM, Chang-Claude J, Slattery ML, Pflugeisen BM, Lin Y, Duggan D, Nan H, Lemire M. et al, Characterization of gene-environment interactions for colorectal cancer susceptibility loci. Cancer Res. 2012 Apr 15;72(8):2036-44. doi: 10.1158/0008-5472.CAN-11-4067. Epub 2012 Feb 24. PMID: 22367214; PMCID: PMC3374720. | Excluded | red and processed meat exposures characterized only as above/below median |
| Järvinen R, Knekt P, Hakulinen T, Rissanen H, Heliövaara M. Dietary fat, cholesterol and colorectal cancer in a prospective study. Br J Cancer. 2001; 85(3): 357-61. | Excluded | exposure is combined red meat |
| Jones, P., Cade, J., Evans, C., Hancock, N., & Greenwood, D. (2018). Does adherence to the World Cancer Research Fund/American Institute of Cancer Research cancer prevention guidelines reduce risk of colorectal cancer in the UK Women’s Cohort Study? *British Journal of Nutrition,* *119*(3), 340-348. doi:10.1017/S0007114517003622 | Excluded | exposure is to adherence to WCRF guidelines |
| Jones RR, DellaValle CT, Weyer PJ, Robien K, Cantor KP, Krasner S, Beane Freeman LE, Ward MH. Ingested nitrate, disinfection by-products, and risk of colon and rectal cancers in the Iowa Women's Health Study cohort. Environ Int. 2019; 126: 242-251. | Excluded | exposure stratification between red and processed is uncertain, same cohort is used in another study with clearer description (Sellers 1998) |
| Kabat GC, Miller AB, Jain M, Rohan TE. A cohort study of dietary iron and heme iron intake and risk of colorectal cancer in women. Br J Cancer. 2007; 97(1): 118-22. | Excluded | exposure is combined red meat |
| Kappeler R, Eichholzer M, Rohrmann S. Meat consumption and diet quality and mortality in NHANES III. Eur J Clin Nutr. 2013; 67(6): 598-606. | Excluded | exposure is combined red meat |
| Kato I, Akhmedkhanov A, Koenig K, et al. Prospective study of diet and female colorectal cancer: the New York University Women’s Health Study. Nutr Cancer 1997;28:276-81. | Excluded | exposure is likely to combined red meat, stratification between red and processed includes ham/sausages together |
| Kesse E, Clavel-Chapelon F, Boutron-Ruault MC (2006). Dietary patterns and risk of colorectal tumors: a cohort of French women of the national education system (E3N). Am J Epidemiol 164:1085–1093. | Excluded | exposure is dietary pattern (vegetarian or not), no meat percentile characterization |
| Key, T., Fraser, G., Thorogood, M., Appleby, P., Beral, V., Reeves, G., . . . McPherson, K. (1998). Mortality in vegetarians and non-vegetarians: A collaborative analysis of 8300 deaths among 76,000 men and women in five prospective studies. Public Health Nutrition, 1(1), 33-41. doi:10.1079/PHN19980006 | Excluded | exposure is vegetarian/non vegetarian, i.e., total meat consumption |
| Key TJ, Appleby PN, Spencer EA, Travis RC, Roddam AW, Allen NE. Cancer incidence in vegetarians: results from the European Prospective Investigation into Cancer and Nutrition (EPIC-Oxford). Am J Clin Nutr. 2009 May;89(5):1620S-1626S. doi: 10.3945/ajcn.2009.26736M. Epub 2009 Mar 11. PMID: 19279082. | Excluded | exposure is vegetarian/non vegetarian, i.e., total meat consumption |
| Key TJ, Appleby PN, Crowe FL, Bradbury KE, Schmidt JA, Travis RC. Cancer in British vegetarians: updated analyses of 4998 incident cancers in a cohort of 32,491 meat eaters, 8612 fish eaters, 18,298 vegetarians, and 2246 vegans. Am J Clin Nutr. 2014;100 Suppl 1(1):378S-85S. doi:10.3945/ajcn.113.071266 | Excluded | exposure is vegetarian/non vegetarian, i.e., total meat consumption |
| Khan, M.M.H., Goto, R., Kobayashi, K., Suzumura, S., Nagata, Y., Sonoda, T., Sakauchi, F., Washio, M. and Mori, M., 2004. Dietary habits and cancer mortality among middle aged and older Japanese living in hokkaido, Japan by cancer sites and sex. Asian Pacific Journal of Cancer Prevention, 5(1), pp.58-65. | Excluded | exposure is to a subset of total red meat, processed not completely separated (nor some poultry?) |
| Kim J, Park S, Nam BH (2011). The risk of colorectal cancer is associated with the frequency of meat consumption in a population-based cohort in Korea. Asian Pac J Cancer Prev, 12, 2371-6. | Excluded | exposure is to total meat |
| Knekt P, Steineck G, Järvinen R, Hakulinen T, Aromaa A. Intake of fried meat and risk of cancer: a follow-up study in Finland. Int J Cancer. 1994 Dec 15;59(6):756-60. doi: 10.1002/ijc.2910590608. PMID: 7989114. | Excluded | exposure is to fried meat, risks for red or processed not reported separately |
| Knekt P, Järvinen R, Dich J, Hakulinen T. Risk of colorectal and other gastro-intestinal cancers after exposure to nitrate, nitrite and N-nitroso compounds: a follow-up study. Int J Cancer. 1999 Mar 15;80(6):852-6. doi: 10.1002/(sici)1097-0215(19990315)80:6<852::aid-ijc9>3.0.co;2-s. PMID: 10074917. | Excluded | meat exposure may include poultry, and cohort already included via GBD-cited study |
| Knuppel A, Papier K, Fensom GK, Appleby PN, Schmidt JA, Tong TYN, Travis RC, Key TJ, Perez-Cornago A. Meat intake and cancer risk: prospective analyses in UK Biobank. Int J Epidemiol. 2020 Oct 1;49(5):1540-1552. doi: 10.1093/ije/dyaa142. PMID: 32814947. | Included |  |
| Ko K-P, Park SK, Yang JJ, et al (2013). Intake of soy products and other foods and gastric cancer risk: a prospective study. J Epidemiol, 23, 337. | Excluded | exposure is to total meat |
| Kojima M, Wakai K, Tamakoshi K, et al. Diet and colorectal cancer mortality: results from the Japan Collaborative Cohort Study, Nutr Cancer, 2004, vol. 50 (pg. 23-32) | Excluded | exposure is to total meat |
| Larsson SC, Rafter J, Holmberg L, Bergkvist L, Wolk A. Red meat consumption and risk of cancers of the proximal colon, distal colon and rectum: the Swedish Mammography Cohort. Int J Cancer. 2005; 113(5): 829–34. | Included |  |
| Lee S-A, Shu XO, Yang G, Li H, Gao Y-T, Zheng W. Animal origin foods and colorectal cancer risk: a report from the Shanghai Women’s Health Study. Nutr Cancer. 2009; 61(2): 194–205. | Excluded | exposure is combined red meat |
| Lee JE, McLerran DF, Rolland B, Chen Y, Grant EJ, Vedanthan R, Inoue M, Tsugane S, et al. Meat intake and cause-specific mortality: a pooled analysis of Asian prospective cohort studies. Am J Clin Nutr. 2013; 98(4): 1032-41. | Excluded | Outcome is all cancer mortality |
| Levi, F., Pasche, C., La Vecchia, C., Lucchini, F. and Franceschi, S., 1999. Food groups and colorectal cancer risk. British journal of cancer, 79(7), pp.1283-1287. | Excluded | pork grouped with processed meat, and is a case-control |
| Lin J, Zhang SM, Cook NR, Lee I-M, Buring JE. Dietary fat and fatty acids and risk of colorectal cancer in women. Am J Epidemiol. 2004; 160(10): 1011–22. | Included (PM only) |  |
| Lüchtenborg, Margreet, Matty P. Weijenberg, Anton FPM De Goeij, Petra A. Wark, Mirian Brink, Guido MJM Roemen, Marjolein HFM Lentjes et al. "Meat and fish consumption, APC gene mutations and hMLH1 expression in colon and rectal cancer: a prospective cohort study (The Netherlands)." Cancer Causes & Control 16 (2005): 1041-1054. | Excluded | Nested case control, but cohort is already included (Netherlands cohort) |
| Martínez, M.E., Jacobs, E.T., Ashbeck, E.L., Sinha, R., Lance, P., Alberts, D.S. and Thompson, P.A., 2007. Meat intake, preparation methods, mutagens and colorectal adenoma recurrence. Carcinogenesis, 28(9), pp.2019-2027. | Excluded | exposure to red meat may contain processed meat, definitions are unclear. |
| Mathew, A., Peters, U., Chatterjee, N., Kulldorff, M. and Sinha, R., 2004. Fat, fiber, fruits, vegetables, and risk of colorectal adenomas. International journal of cancer, 108(2), pp.287-292. | Excluded | exposure is to fat from red meat |
| Mehta SS, Arroyave WD, Lunn RM, Park YM, Boyd WA, Sandler DP. A Prospective Analysis of Red and Processed Meat Consumption and Risk of Colorectal Cancer in Women. Cancer Epidemiol Biomarkers Prev. 2020 Jan;29(1):141-150. doi: 10.1158/1055-9965.EPI-19-0459. Epub 2019 Oct 1. PMID: 31575555; PMCID: PMC6954313. | Included |  |
| Mejborn H, Møller SP, Thygesen LC, Biltoft-Jensen A. Dietary Intake of Red Meat, Processed Meat, and Poultry and Risk of Colorectal Cancer and All-Cause Mortality in the Context of Dietary Guideline Compliance. *Nutrients*. 2021; 13(1):32. https://doi.org/10.3390/nu13010032 | Included |  |
| Norat T, Bingham S, Ferrari P, Slimani N, Jenab M, Mazuir M, Overvad K, et al. Meat, fish, and colorectal cancer risk: the European Prospective Investigation into cancer and nutrition. J Natl Cancer Inst. 2005; 97(12): 906–16. | Excluded | a more recent study uses the same cohort (Ward 2017) |
| Nowell, S., Coles, B., Sinha, R., MacLeod, S., Ratnasinghe, D.L., Stotts, C., Kadlubar, F.F., Ambrosone, C.B. and Lang, N.P., 2002. Analysis of total meat intake and exposure to individual heterocyclic amines in a case-control study of colorectal cancer: contribution of metabolic variation to risk. Mutation Research/Fundamental and Molecular Mechanisms of Mutagenesis, 506, pp.175-185. | Excluded | Exposure is to well-done meats (combined processed and red) |
| Oba S, Shimizu N, Nagata C, Shimizu H, Kametani M, Takeyama N, Ohnuma T, Matsushita S. The relationship between the consumption of meat, fat, and coffee and the risk of colon cancer: a prospective study in Japan. Cancer Lett. 2006; 244(2): 260-7. | Included |  |
| Ollberding NJ, Wilkens LR, Henderson BE, Kolonel LN, Le Marchand L. Meat consumption, heterocyclic amines and colorectal cancer risk: the Multiethnic Cohort Study. Int J Cancer. 2012; 131(7): E1125-33. | Included |  |
| Orlich MJ, Singh PN, Sabaté J, Fan J, Sveen L, Bennett H, Knutsen SF, Beeson WL, Jaceldo-Siegl K, Butler TL, Herring RP, Fraser GE. Vegetarian dietary patterns and the risk of colorectal cancers. JAMA Intern Med. 2015 May;175(5):767-76. doi: 10.1001/jamainternmed.2015.59. PMID: 25751512; PMCID: PMC4420687. | Excluded | exposure was characterized as diet type (vegetarian etc) |
| Pan A, Sun Q, Bernstein AM, Schulze MB, Manson JE, Stampfer MJ, Willett WC, Hu FB. Red meat consumption and mortality: results from 2 prospective cohort studies. Arch Intern Med. 2012; 172(7): 555-63. | Excluded | Outcome is all cancer mortality |
| Parr CL, Hjartåker A, Lund E, Veierød MB. Meat intake, cooking methods and risk of proximal colon, distal colon and rectal cancer: the Norwegian Women and Cancer (NOWAC) cohort study. Int J Cancer. 2013; 133(5): 1153-63. | Included |  |
| Phillips RL, Snowdon DA. Dietary relationships with fatal colorectal cancer among Seventh-Day Adventists. J Natl Cancer Inst. 1985 Feb;74(2):307-17. PMID: 3856044. | Excluded | exposure is to total meat, cohort already included via later study |
| Pietinen P, Malila N, Virtanen M, Hartman TJ, Tangrea JA, Albanes D, Virtamo J. Diet and risk of colorectal cancer in a cohort of Finnish men. Cancer Causes Control. 1999; 10(5): 387–96. | Included (PM only) |  |
| Rada-Fernandez de Jauregui D, Evans CEL, Jones P, Greenwood DC, Hancock N, Cade JE. Common dietary patterns and risk of cancers of the colon and rectum: Analysis from the United Kingdom Women's Cohort Study (UKWCS). Int J Cancer. 2018 Aug 15;143(4):773-781. doi: 10.1002/ijc.31362. Epub 2018 Apr 1. Erratum in: Int J Cancer. 2021 Apr 15;148(8):E8. PMID: 29516512; PMCID: PMC6055763. | Excluded | exposure is to combined red meat, and not quantified (only exposed/not) |
| Rohrmann S, Overvad K, Bueno-de-Mesquita HB, Jakobsen MU, Egeberg R, Tjønneland A, et al., Meat consumption and mortality-results from the European Prospective Investigation into Cancer and Nutrition. BMC Med. 2013; 11: 63. | Excluded | Outcome is all cancer mortality |
| Ruder EH, Thiébaut AC, Thompson FE, Potischman N, Subar AF, Park Y, Graubard BI, Hollenbeck AR, Cross AJ. Adolescent and mid-life diet: risk of colorectal cancer in the NIH-AARP Diet and Health Study. Am J Clin Nutr. 2011 Dec;94(6):1607-19. doi: 10.3945/ajcn.111.020701. Epub 2011 Nov 9. PMID: 22071715; PMCID: PMC3252554. | Excluded | red meat category contains processed meat products, and another study uses this cohort |
| Sanjoaquin MA, Appleby PN, Thorogood M, Mann JI, Key TJ. Nutrition, lifestyle and colorectal cancer incidence: a prospective investigation of 10998 vegetarians and non-vegetarians in the United Kingdom. Br J Cancer. 2004;90(1):118-121. doi:10.1038/sj.bjc. | Excluded | exposure is vegetarian/non vegetarian, i.e., total meat consumption |
| Sato, Y., Nakaya, N., Kuriyama, S., Nishino, Y., Tsubono, Y., & Tsuji, I. (2006). Meat consumption and risk of colorectal cancer in Japan: The Miyagi Cohort Study. *European Journal of Cancer Prevention*, *15*(3), 211–218. http://www.jstor.org/stable/45051603 | Included (PM only) |  |
| Sellers TTA, Bazyk AAE, Bostick RRM, et al. Diet and risk of colon cancer in a large prospective study of older women: an analysis stratified on family history (Iowa, United States). Cancer Causes Control 1998;9:357-67. | Included (PM only) |  |
| Sharma I, Roebothan B, Zhu Y, Woodrow J, Parfrey PS, Mclaughlin JR, Wang PP. Hypothesis and data-driven dietary patterns and colorectal Cancer survival: findings from Newfoundland and Labrador colorectal Cancer cohort. Nutr J. 2018 May 25;17(1):55. doi: 10.1186/s12937-018-0362-x. PMID: 29793493; PMCID: PMC5968482. | Excluded | outcome is mortality/recurrence/metastasis, not incidence |
| Singh PN, Fraser GE. Dietary risk factors for colon cancer in a low-risk population. Am J Epidemiol. 1998; 148(8): 761–74. | Excluded | exposure is combined red meat |
| Sinha R, Cross AJ, Graubard BI, Leitzmann MF, Schatzkin A. Meat intake and mortality: a prospective study of over half a million people. Arch Intern Med. 2009; 169(6): 562–71. | Excluded | Outcome is all cancer mortality |
| Snowdon, DA (1988) Animal product consumption and mortality because of all causes combined, coronary heart disease, stroke, diabetes, and cancer in Seventh-day Adventists. Am J Clin Nutr 48, 739–748.CrossRefGoogle ScholarPubMed | Excluded | exposure is to total meat |
| Steinmetz, K.A. and Potter, J.D. (1993), Food-group consumption and colon cancer in the adelaide case-control study. II. Meat, poultry, seafood, dairy foods and eggs. Int. J. Cancer, 53: 720-727. https://doi-org.ezproxy2.library.colostate.edu/10.1002/ijc.2910530503 | Excluded | exposure is to combined red meat |
| Takachi R, Tsubono Y, Baba K, Inoue M, Sasazuki S, Iwasaki M, Tsugane S, Japan Public Health Center-Based Prospective Study Group. Red meat intake may increase the risk of colon cancer in Japanese, a population with relatively low red meat consumption. Asia Pac J Clin Nutr. 2011; 20(4): 603-12. | Included (PM only) |  |
| Takata Y, Shu XO, Gao YT, Li H, Zhang X, Gao J, Cai H, Yang G, Xiang YB, Zheng W. Red meat and poultry intakes and risk of total and cause-specific mortality: results from cohort studies of Chinese adults in Shanghai. PLoS One. 2013; 8(2): e56963. | Excluded | exposure is combined red meat |
| Tantamango YM, Knutsen SF, Beeson WL, Fraser G, Sabate J. Foods and food groups associated with the incidence of colorectal polyps: the Adventist Health Study. Nutr Cancer. 2011;63(4):565-72. doi: 10.1080/01635581.2011.551988. PMID: 21547850; PMCID: PMC3427008. | Excluded | exposure is to combined red meat |
| Tavani A, La Vecchia C, Gallus S, Lagiou P, Trichopoulos D, Levi F, Negri E. Red meat intake and cancer risk: a study in Italy. Int J Cancer. 2000 May 1;86(3):425-8. doi: 10.1002/(sici)1097-0215(20000501)86:3<425::aid-ijc19>3.0.co;2-s. PMID: 10760833. | Excluded | case control study from multiple separate studies |
| Terry P, Hu FB, Hansen H, Wolk A. Prospective study of major dietary patterns and colorectal cancer risk in women. Am J Epidemiol. 2001 Dec 15;154(12):1143-9. doi: 10.1093/aje/154.12.1143. PMID: 11744520. | Excluded | relative risks are only reported for dietary scores, cohort is included via later study |
| Tiemersma EW, Kampman E, Bueno de Mesquita HB, Bunschoten A, van Schothorst EM, Kok FJ, Kromhout D. Meat consumption, cigarette smoking, and genetic susceptibility in the etiology of colorectal cancer: results from a Dutch prospective study. Cancer Causes Control. 2002; 13(4): 383–93. | Included (RM only) |  |
| van den Brandt PA. Red meat, processed meat, and other dietary protein sources and risk of overall and cause-specific mortality in The Netherlands Cohort Study. Eur J Epidemiol. 2019; 34(4): 351-369. | Excluded | Outcome is all cancer mortality |
| Vinikoor LC, Satia JA, Schroeder JC, Millikan RC, Martin CF, Ibrahim JG, Sandler RS. Associations between trans fatty acid consumption and colon cancer among Whites and African Americans in the North Carolina colon cancer study I. Nutr Cancer. 2009; 61(4): 427–36. | Excluded | Exposures is trans-fatty acid consumption, not red meat/processed meat |
| Vulcan A, Ericson U, Manjer J, Ohlsson B. A colorectal cancer diet quality index is inversely associated with colorectal cancer in the Malmö diet and cancer study. Eur J Cancer Prev. 2018. | Included (PM only) |  |
| Wada K, Oba S, Tsuji M, Tamura T, Konishi K, Goto Y, Mizuta F, Koda S, Hori A, Tanabashi S, Matsushita S, Tokimitsu N, Nagata C. Meat consumption and colorectal cancer risk in Japan: The Takayama study. Cancer Sci. 2017; 108(5): 1065-1070. | Included |  |
| Ward HA, Norat T, Overvad K, Dahm CC, Bueno-de-Mesquita HB, et al. Pre-diagnostic meat and fibre intakes in relation to colorectal cancer survival in the European Prospective Investigation into Cancer and Nutrition. Br J Nutr. 2016; 116(2): 316-25. | Included |  |
| Wei EK, Giovannucci E, Wu K, Rosner B, Fuchs CS, Willett WC, Colditz GA. Comparison of risk factors for colon and rectal cancer. Int J Cancer. 2004; 108(3): 433-42. | Included |  |
| Wie G-A, Cho Y-A, Kang H, Ryu K-A, Yoo M-K, Kim Y-A, Jung K-W, Kim J, Lee J-H, Joung H. Red meat consumption is associated with an increased overall cancer risk: a prospective cohort study in Korea. Br J Nutr. 2014; 112(2): 238–47. | Excluded | exposure is combined red meat |
| Willett WC, Stampfer MJ, Colditz GA, et al. Relation of meat, fat, and fiber intake to the risk of colon cancer in a prospective study among women. N Engl J Med 1990;323:1664-72. | Excluded | cohort is included through a more recent study |
| Kana Wu, Diane Feskanich, Charles S. Fuchs, Walter C. Willett, Bruce W. Hollis, Edward L. Giovannucci, A Nested Case–Control Study of Plasma 25-Hydroxyvitamin D Concentrations and Risk of Colorectal Cancer, JNCI: Journal of the National Cancer Institute, Volume 99, Issue 14, 18 July 2007, Pages 1120–1129, https://doi-org.ezproxy2.library.colostate.edu/10.1093/jnci/djm038 | Excluded | exposure is serum plasma, RR not reported for meat consumption |
| Zhu Y, Wu H, Wang PP, Savas S, Woodrow J, Wish T, Jin R, Green R, Woods M, Roebothan B, Buehler S, Dicks E, McLaughlin JR, Campbell PT, Parfrey PS. Dietary patterns and colorectal cancer recurrence and survival: a cohort study. BMJ Open. 2013 Feb 7;3(2):e002270. doi: 10.1136/bmjopen-2012-002270. PMID: 23396503; PMCID: PMC3586110. | Excluded | outcome is CRC recurrence rather than initial diagnosis |

Supplemental Table 3: Relative risk estimated per gram for DRMA (mean and 95% Confidence interval) for models fit only to low-consumer data for processed red meat consumption and colorectal cancer, omitting Ward et al 2016 as outcome is mortality rather than diagnosis. Models are the empirical DR and the 2-stage RCS DR model.

| grams/  day | Empirical DR LD | | 2-stage RCS LD | | Empirical DR | | 2-stage RCS All | |
| --- | --- | --- | --- | --- | --- | --- | --- | --- |
|  | Mean | 95% CI | Mean | 95% CI | Mean | 95% CI | Mean | 95% CI |
| 0 | 1.0381 | (0.9409-1.1454) | 1.001 | (0.989-1.0132) | 1.0037 | (0.9761-1.0322) | 1 | (1-1) |
| 1 | 1.0483 | (0.9885-1.1117) | 1.0021 | (0.9783-1.0264) | 0.9877 | (0.9564-1.0201) | 1.0056 | (1.0024-1.0088) |
| 2 | 0.9883 | (0.9583-1.0193) | 1.0033 | (0.9683-1.0395) | 0.9954 | (0.9635-1.0283) | 1.0111 | (1.0048-1.0176) |
| 3 | 1.0029 | (0.96-1.0477) | 1.0047 | (0.9594-1.0521) | 1.0019 | (0.9902-1.0138) | 1.0167 | (1.0071-1.0264) |
| 4 | 1.0015 | (0.9832-1.0202) | 1.0064 | (0.9517-1.0642) | 1.009 | (0.9935-1.0247) | 1.0224 | (1.0095-1.0353) |
| 5 | 1.0105 | (0.9799-1.0421) | 1.0084 | (0.9456-1.0754) | 1.022 | (0.9987-1.0458) | 1.028 | (1.012-1.0443) |
| 6 | 1.0154 | (0.9758-1.0565) | 1.0108 | (0.9413-1.0855) | 1.0182 | (0.989-1.0482) | 1.0336 | (1.0144-1.0532) |
| 7 | 1.0199 | (0.9749-1.067) | 1.0138 | (0.9391-1.0944) | 1.0032 | (0.9858-1.0209) | 1.0392 | (1.0168-1.0621) |
| 8 | 1.0235 | (0.9752-1.0741) | 1.0174 | (0.9393-1.1019) | 0.9936 | (0.979-1.0084) | 1.0448 | (1.0193-1.071) |
| 9 | 1.0259 | (0.9758-1.0786) | 1.0216 | (0.9419-1.1079) | 1.001 | (0.9877-1.0145) | 1.0504 | (1.0217-1.0798) |
| 10 | 1.0294 | (0.9783-1.0831) | 1.0264 | (0.9467-1.1128) | 1.0018 | (0.9922-1.0116) | 1.0559 | (1.0242-1.0886) |
| 11 | 1.0364 | (0.9844-1.0912) | 1.0317 | (0.953-1.1169) | 1.0046 | (0.9875-1.022) | 1.0614 | (1.0267-1.0973) |
| 12 | 1.0424 | (0.9897-1.098) | 1.0375 | (0.9604-1.1209) | 1.0087 | (0.9857-1.0323) | 1.0669 | (1.0292-1.1059) |
| 13 | 1.0478 | (0.9933-1.1053) | 1.0438 | (0.9682-1.1252) | 1.0143 | (0.9863-1.043) | 1.0723 | (1.0318-1.1143) |
| 14 | 1.0502 | (0.9939-1.1096) | 1.0504 | (0.9759-1.1305) | 1.0209 | (0.9891-1.0538) | 1.0776 | (1.0343-1.1227) |
| 15 | 1.0535 | (0.9913-1.1196) | 1.0573 | (0.9826-1.1375) | 1.0285 | (0.9934-1.0647) | 1.0829 | (1.0369-1.1308) |
| 16 | 1.0575 | (0.9923-1.1269) | 1.0644 | (0.988-1.1466) | 1.0372 | (0.9988-1.077) | 1.088 | (1.0395-1.1388) |
| 17 | 1.0622 | (0.9917-1.1378) | 1.0716 | (0.9916-1.1581) | 1.0462 | (1.0047-1.0895) | 1.0931 | (1.0422-1.1466) |
| 18 | 1.0614 | (0.9618-1.1712) | 1.079 | (0.9935-1.1719) | 1.0687 | (1.0168-1.1232) | 1.0981 | (1.0448-1.1542) |
| 19 | 1.0222 | (0.8746-1.1946) | 1.0864 | (0.9938-1.1876) | 1.0772 | (1.0224-1.1349) | 1.103 | (1.0475-1.1615) |
| 20 | 1.0392 | (0.8371-1.29) | 1.0864 | (0.9938-1.1876) | 1.0857 | (1.0282-1.1465) | 1.1078 | (1.0502-1.1686) |
| 21 | 1.0392 | (0.8371-1.29) | 1.0864 | (0.9938-1.1876) | 1.0857 | (1.0282-1.1465) | 1.1078 | (1.0502-1.1686) |

Supplemental Table 4: Relative risk estimated per gram for DRMA (mean and 95% Confidence interval) for models fit only to low-consumer data for unprocessed red meat consumption and colorectal cancer, omitting Ward et al 2016 as outcome is mortality rather than diagnosis. Models are the empirical DR and the 2-stage RCS DR model.

| grams/  day | Empirical DR LD | | 2-stage RCS LD | | Empirical DR | | | 2-stage RCS All | |
| --- | --- | --- | --- | --- | --- | --- | --- | --- | --- |
|  | Mean | 95% CI | Mean | 95% CI | | Mean | 95% CI | Mean | 95% CI |
| 0 | 1.000 | (1-1) | 1 | (1-1) | | 1.0570 | (0.9988-1.1188) | 1 | (1-1) |
| 1 | 1.002 | (0.9972-1.0059) | 1.0015 | (0.9972-1.0059) | | 1.0570 | (0.997-1.1727) | 1.0024 | (1.0004-1.0044) |
| 2 | 1.003 | (0.9943-1.0118) | 1.0030 | (0.9943-1.0118) | | 1.0570 | (0.9961-1.2014) | 1.0048 | (1.0009-1.0087) |
| 3 | 1.005 | (0.9915-1.0177) | 1.0045 | (0.9915-1.0177) | | 1.0812 | (0.9947-1.2201) | 1.0072 | (1.0013-1.0132) |
| 4 | 1.006 | (0.9887-1.0237) | 1.0060 | (0.9887-1.0237) | | 1.0939 | (0.9934-1.2323) | 1.0096 | (1.0018-1.0176) |
| 5 | 1.008 | (0.9859-1.0297) | 1.0075 | (0.9859-1.0297) | | 1.1016 | (0.9871-1.2201) | 1.0121 | (1.0022-1.022) |
| 6 | 1.009 | (0.9831-1.0357) | 1.0090 | (0.9831-1.0357) | | 1.1064 | (0.9943-1.0125) | 1.0145 | (1.0027-1.0265) |
| 7 | 1.011 | (0.9804-1.0417) | 1.0105 | (0.9804-1.0417) | | 1.0974 | (0.9898-1.026) | 1.0169 | (1.0031-1.0309) |
| 8 | 1.012 | (0.9777-1.0477) | 1.0120 | (0.9777-1.0477) | | 1.0033 | (0.9865-1.0402) | 1.0194 | (1.0036-1.0354) |
| 9 | 1.014 | (0.975-1.0537) | 1.013 | (0.975-1.0537) | | 1.0077 | (0.9935-1.0089) | 1.0218 | (1.004-1.0399) |
| 10 | 1.015 | (0.9724-1.0596) | 1.0150 | (0.9724-1.0596) | | 1.0130 | (0.9888-1.0184) | 1.0243 | (1.0044-1.0445) |
| 11 | 1.017 | (0.9699-1.0655) | 1.0165 | (0.9699-1.0655) | | 1.0011 | (0.973-1.0245) | 1.0267 | (1.0049-1.049) |
| 12 | 1.018 | (0.9674-1.0713) | 1.0180 | (0.9674-1.0713) | | 1.0035 | (0.9848-1.0281) | 1.0291 | (1.0053-1.0535) |
| 13 | 1.019 | (0.9651-1.077) | 1.0194 | (0.9651-1.077) | | 0.9984 | (0.9801-1.0391) | 1.0316 | (1.0058-1.058) |
| 14 | 1.021 | (0.9628-1.0826) | 1.0209 | (0.9628-1.0826) | | 1.0061 | (0.9948-1.0174) | 1.034 | (1.0062-1.0626) |
| 15 | 1.000 | (0.9535-1.0486) | 1.0223 | (0.9606-1.0881) | | 1.009 | (0.9915-1.0327) | 1.0365 | (1.0067-1.0671) |
| 16 | 1.000 | (0.947-1.0564) | 1.0237 | (0.9585-1.0935) | | 1.0060 | (0.9893-1.0456) | 1.0389 | (1.0071-1.0717) |
| 17 | 1.001 | (0.9419-1.063) | 1.0251 | (0.9565-1.0987) | | 1.0119 | (0.9877-1.0566) | 1.0413 | (1.0076-1.0762) |
| 18 | 1.001 | (0.9377-1.0685) | 1.0265 | (0.9546-1.1038) | | 1.0170 | (1.0008-1.0349) | 1.0438 | (1.0081-1.0807) |
| 19 | 1.013 | (0.9955-1.0312) | 1.0278 | (0.9529-1.1088) | | 1.0215 | (1.0015-1.0374) | 1.0462 | (1.0085-1.0853) |
| 20 | 1.017 | (0.9959-1.0386) | 1.029 | (0.9512-1.1135) | | 1.0176 | (0.995-1.0387) | 1.0486 | (1.009-1.0898) |
| 21 | 1.027 | (0.9926-1.0631) | 1.030 | (0.9498-1.118) | | 1.0192 | (0.9967-1.0101) | 1.051 | (1.0094-1.0943) |
| 22 | 1.033 | (0.9883-1.0791) | 1.0317 | (0.9484-1.1224) | | 1.0166 | (0.9954-1.0175) | 1.0534 | (1.0099-1.0988) |
| 23 | 1.035 | (0.9833-1.0893) | 1.0329 | (0.9472-1.1265) | | 1.0033 | (0.9945-1.0246) | 1.0558 | (1.0104-1.1033) |
| 24 | 1.035 | (0.9782-1.0949) | 1.0341 | (0.9461-1.1304) | | 1.0063 | (0.9964-1.0233) | 1.0582 | (1.0108-1.1078) |
| 25 | 1.033 | (0.9733-1.0973) | 1.0353 | (0.9452-1.1341) | | 1.0094 | (0.9959-1.0345) | 1.0606 | (1.0113-1.1123) |
| 26 | 1.031 | (0.9688-1.0975) | 1.0364 | (0.9444-1.1375) | | 1.0097 | (0.9955-1.0425) | 1.0629 | (1.0118-1.1167) |
| 27 | 1.028 | (0.9648-1.0964) | 1.0375 | (0.9438-1.1406) | | 1.0150 | (0.9951-1.049) | 1.0653 | (1.0122-1.1211) |
| 28 | 1.026 | (0.9614-1.0948) | 1.0385 | (0.9433-1.1435) | | 1.0186 | (0.9947-1.0551) | 1.0676 | (1.0127-1.1255) |
| 29 | 1.024 | (0.9586-1.0933) | 1.0395 | (0.9429-1.1462) | | 1.0216 | (0.9944-1.0603) | 1.0699 | (1.0132-1.1299) |
| 30 | 1.022 | (0.9566-1.0923) | 1.0405 | (0.9427-1.1485) | | 1.0244 | (1.0015-1.0193) | 1.0722 | (1.0137-1.1342) |
| 31 | 1.021 | (0.9551-1.092) | 1.0414 | (0.9427-1.1506) | | 1.0268 | (1.0043-1.0375) | 1.0745 | (1.0141-1.1385) |
| 32 | 1.021 | (0.954-1.0922) | 1.0423 | (0.9428-1.1524) | | 1.0103 | (1.0067-1.0525) | 1.0768 | (1.0146-1.1428) |
| 33 | 1.021 | (0.9532-1.0927) | 1.0431 | (0.943-1.154) | | 1.0207 | (1.0084-1.0641) | 1.0791 | (1.0151-1.147) |
| 34 | 1.020 | (0.9524-1.0934) | 1.0439 | (0.9433-1.1553) | | 1.029 | (1.0094-1.073) | 1.0813 | (1.0156-1.1512) |
| 35 | 1.020 | (0.9518-1.0941) | 1.0446 | (0.9436-1.1565) | | 1.0358 | (0.9865-1.0679) | 1.0835 | (1.0161-1.1554) |
| 36 | 1.020 | (0.9511-1.0946) | 1.0453 | (0.944-1.1575) | | 1.0407 | (0.9839-1.0759) | 1.0857 | (1.0166-1.1595) |
| 37 | 1.020 | (0.9505-1.0949) | 1.0459 | (0.9445-1.1584) | | 1.0264 | (0.9847-1.0807) | 1.0879 | (1.0171-1.1636) |
| 38 | 1.003 | (0.9324-1.0799) | 1.0465 | (0.9449-1.1592) | | 1.0288 | (0.9881-1.0839) | 1.09 | (1.0176-1.1676) |
| 39 | 1.003 | (0.9323-1.0798) | 1.0471 | (0.9452-1.16) | | 1.0315 | (0.9918-1.0867) | 1.0922 | (1.0181-1.1716) |
| 40 | 0.992 | (0.919-1.071) | 1.0476 | (0.9455-1.1608) | | 1.0348 | (0.9941-1.0896) | 1.0943 | (1.0186-1.1756) |
| 41 | 0.991 | (0.9186-1.0699) | 1.0481 | (0.9457-1.1617) | | 1.038 | (0.9952-1.0928) | 1.0963 | (1.0191-1.1794) |
| 42 | 0.990 | (0.9179-1.0683) | 1.0486 | (0.9457-1.1627) | | 1.0407 | (0.9957-1.1015) | 1.0984 | (1.0196-1.1833) |
| 43 | 0.989 | (0.9149-1.0702) | 1.0490 | (0.9456-1.1639) | | 1.0428 | (0.9986-1.1091) | 1.1004 | (1.0201-1.187) |
| 44 | 0.997 | (0.9204-1.081) | 1.0494 | (0.9452-1.1652) | | 1.047 | (0.9995-1.1135) | 1.1024 | (1.0206-1.1907) |
| 45 | 0.994 | (0.9177-1.0774) | 1.0498 | (0.9447-1.1668) | | 1.0523 | (1.0007-1.1186) | 1.1044 | (1.0212-1.1944) |
| 46 | 0.964 | (0.8852-1.0502) | 1.050 | (0.944-1.1685) | | 1.0549 | (1.0023-1.1242) | 1.1063 | (1.0217-1.1979) |
| 47 | 0.962 | (0.8831-1.0487) | 1.0506 | (0.943-1.1705) | | 1.0580 | (1.0042-1.1302) | 1.1082 | (1.0222-1.2014) |
| 48 | 0.960 | (0.8802-1.0475) | 1.0509 | (0.9418-1.1728) | | 1.0615 | (0.997-1.1083) | 1.1101 | (1.0227-1.2049) |
| 49 | 0.958 | (0.8761-1.0466) | 1.0513 | (0.9404-1.1754) | | 1.0653 | (0.9988-1.1151) | 1.1119 | (1.0232-1.2083) |
| 50 | 0.955 | (0.8712-1.046) | 1.0516 | (0.9387-1.1782) | | 1.0511 | (0.9982-1.1217) | 1.1137 | (1.0238-1.2116) |
| 51 | 0.806 | (0.5078-1.2787) | 1.0520 | (0.9369-1.1813) | | 1.055 | (0.9995-1.1271) | 1.1155 | (1.0243-1.2148) |
| 52 | 0.805 | (0.5085-1.2736) | 1.0523 | (0.9348-1.1846) | | 1.0581 | (1.0008-1.132) | 1.1172 | (1.0248-1.2179) |
| 53 | 0.804 | (0.5092-1.2685) | 1.0526 | (0.9326-1.1882) | | 1.0613 | (1.0021-1.1366) | 1.1189 | (1.0253-1.221) |
| 54 | 0.600 | (0.3795-0.9487) | 1.0530 | (0.9302-1.1921) | | 1.064 | (1.0042-1.1476) | 1.1205 | (1.0259-1.2239) |
| 55 | 0.600 | (0.3795-0.9487) | 1.0533 | (0.9276-1.1961) | | 1.0672 | (1.0042-1.1476) | 1.1221 | (1.0264-1.2268) |

Supplemental Table 5: Regression coefficients and summary statistics on multivariate MA of lowest consumer groups above the baseline consumption, with a covariate of use of non-consumer baseline relative to mixture of consumers and non-consumers in the baseline.

|  | Variable | Coefficients with 95% CI | Two-sided p-value | One-sided p-value | τ^2^ (SE) | I^2^ |
| --- | --- | --- | --- | --- | --- | --- |
| PM | Consumption (g/day) | 1.003 (0.999-1.008) | 0.1559 | 0.0780 | 0 (0.0032) | 0.00% |
|  | Non-consumer baseline | 0.998 (0.855-1.159) | 0.9373 | 0.4687 |  |  |
| RM | Consumption (g/day) | 1.00 (0.999-1.004) | 0.2993 | 0.1597 | 0 (0.0036) | 0.01% |
|  | Non-consumer baseline | 1.28 (0.993-1.661) | 0.0560 | 0.0280 |  |  |


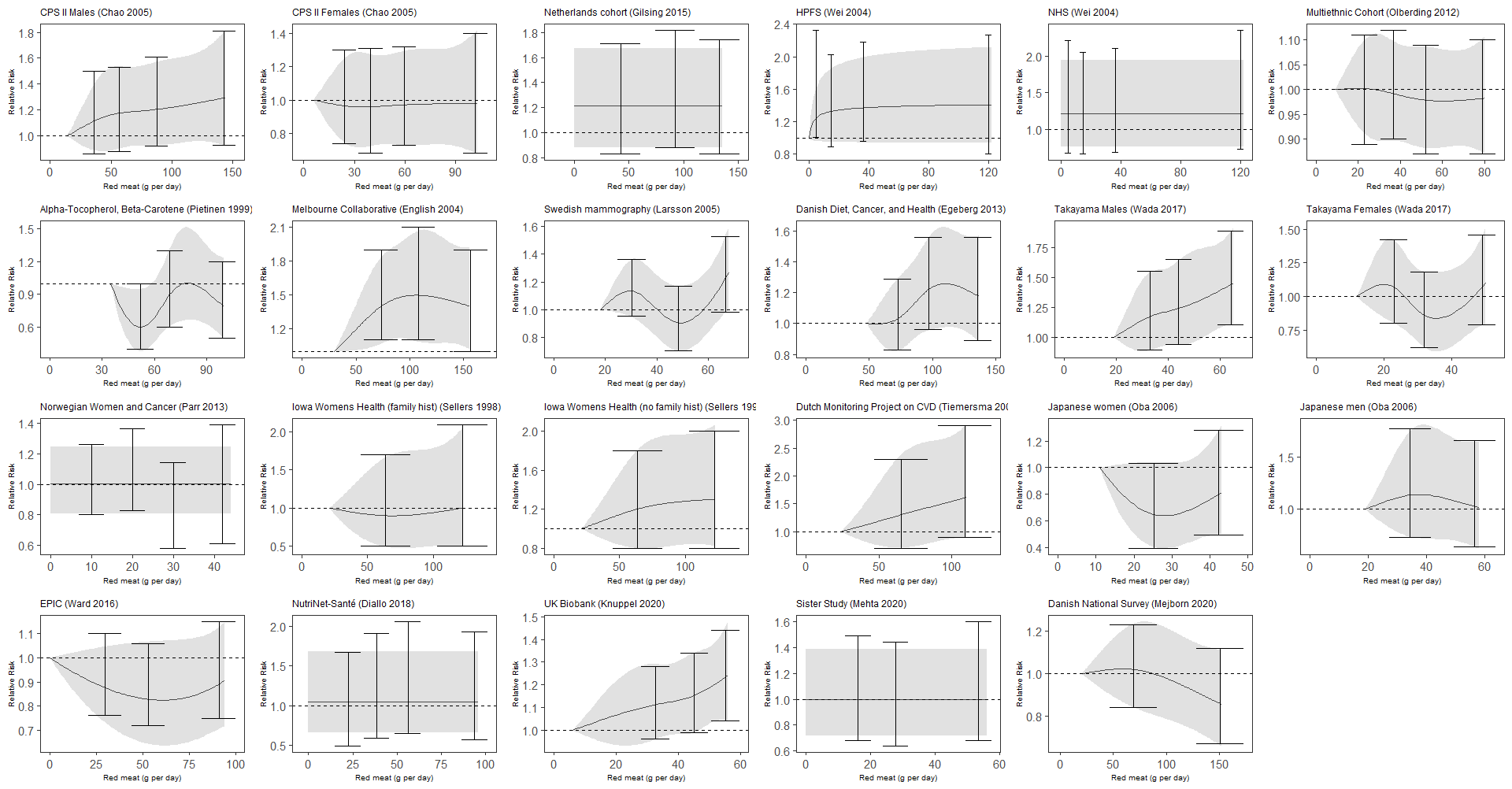


Supplemental Figure 3: Best fitting DR models for individual studies of unprocessed red meat and CRC at all consumption levels within each study, for use in empirical DRMA


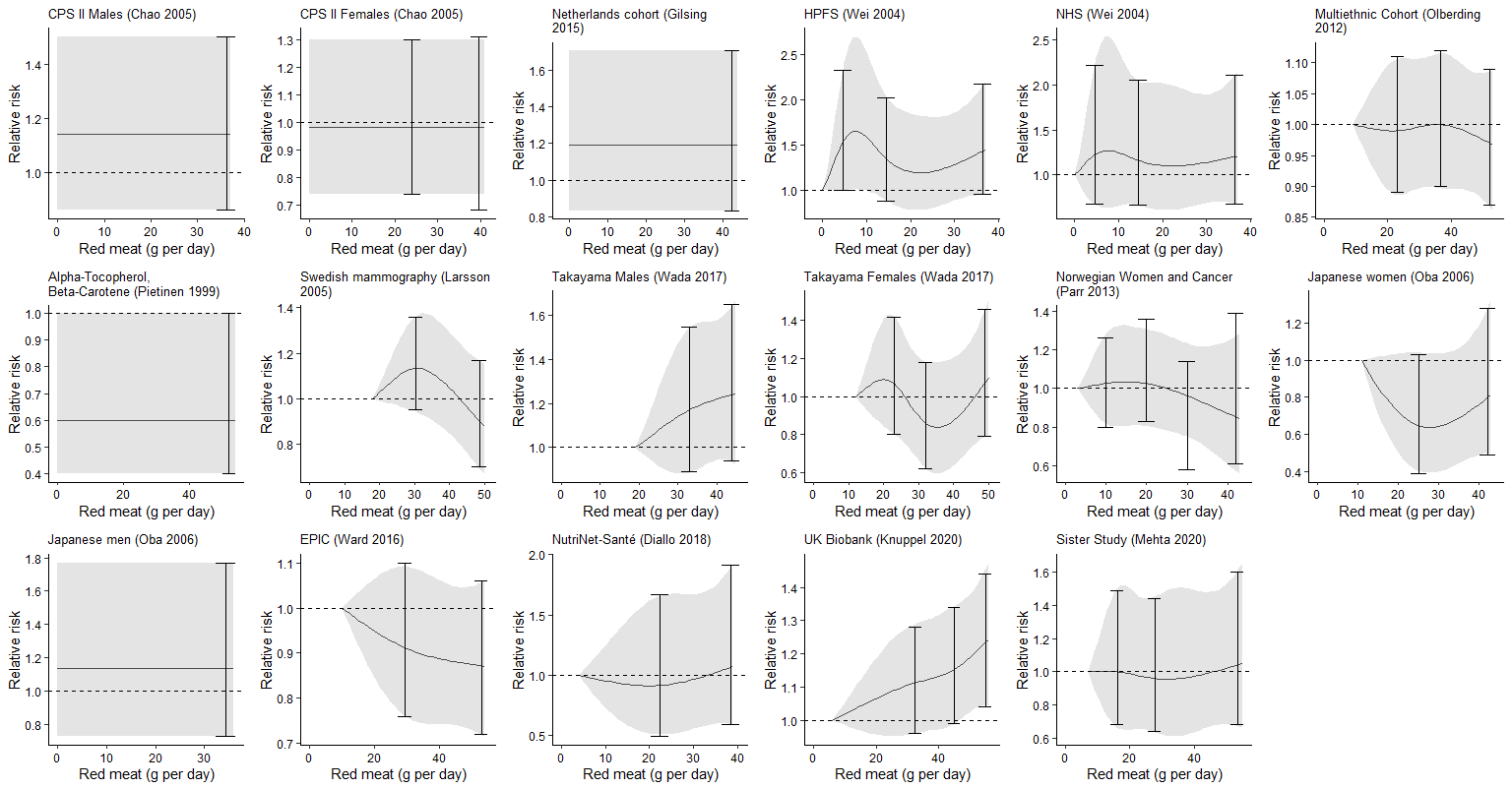


Supplemental Figure 4: Best fitting DR models for individual studies of unprocessed red meat and CRC below 56 grams per day. within each study, for use in empirical DRMA


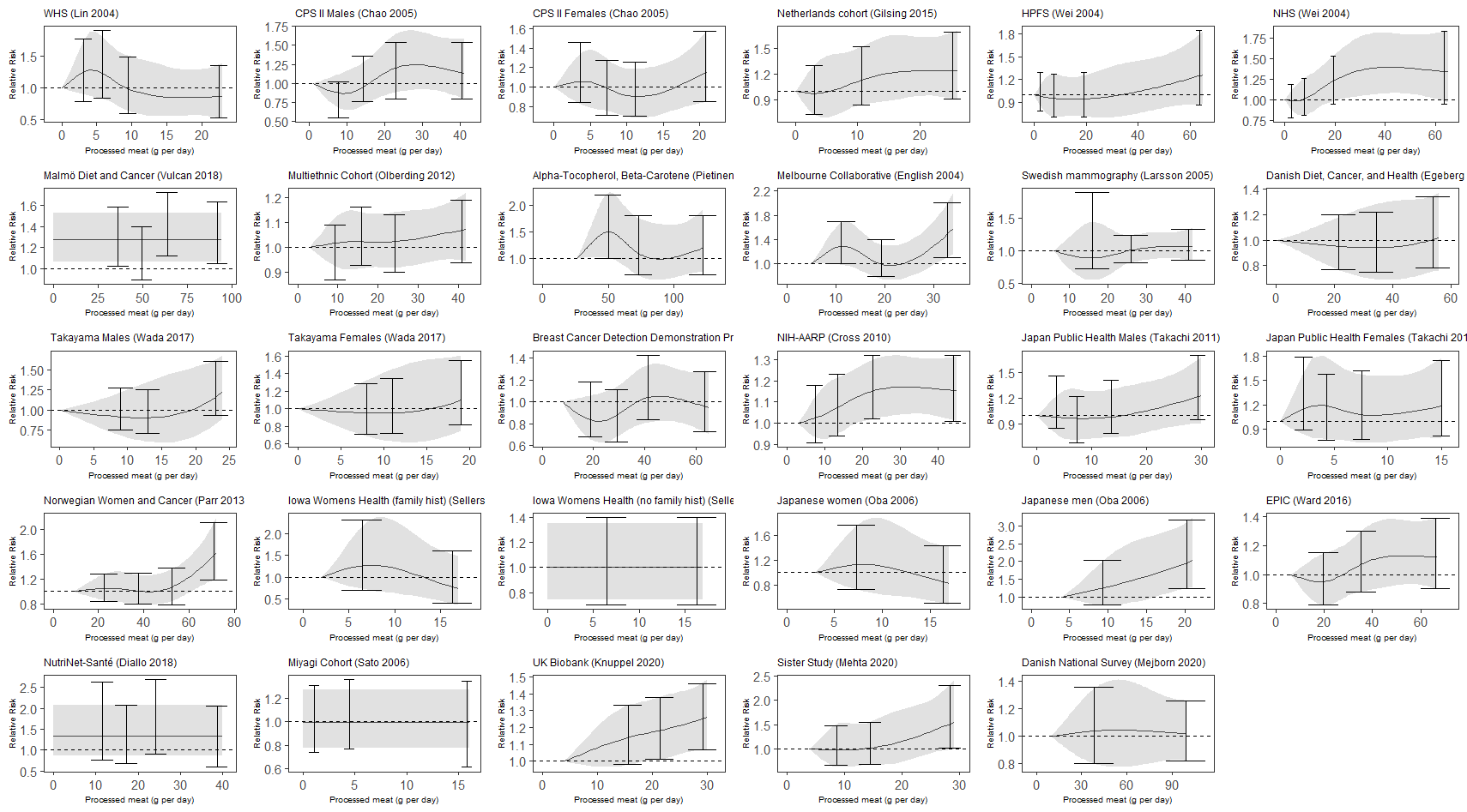


Supplemental Figure 5: Best fitting DR models for individual studies of processed meat and CRC at all consumption levels within each study, for use in empirical DRMA


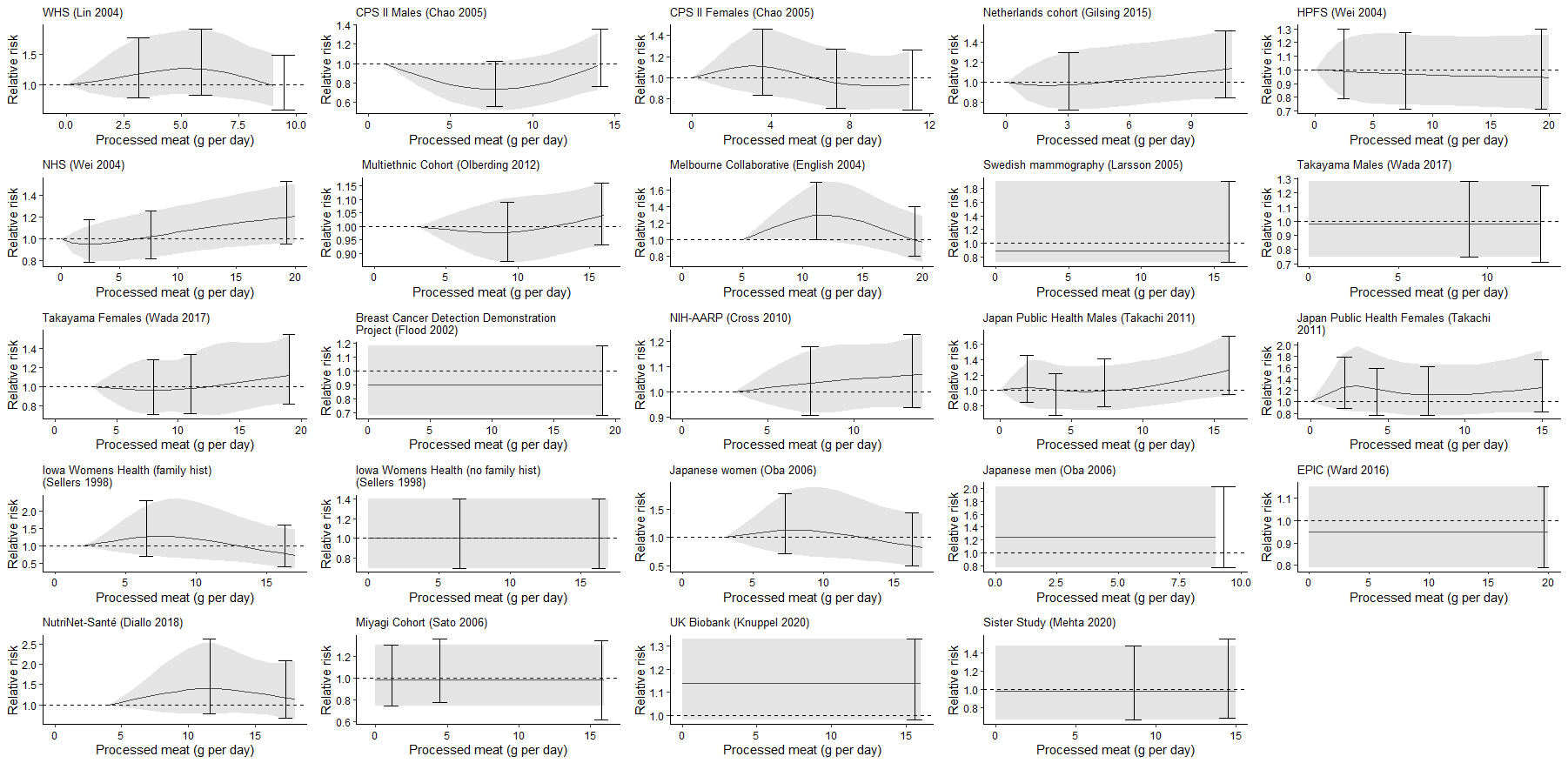


Supplemental Figure 6: Best fitting DR models for individual studies of processed meat and CRC below 21 grams of consumption per day, within each study, for use in empirical DRMA


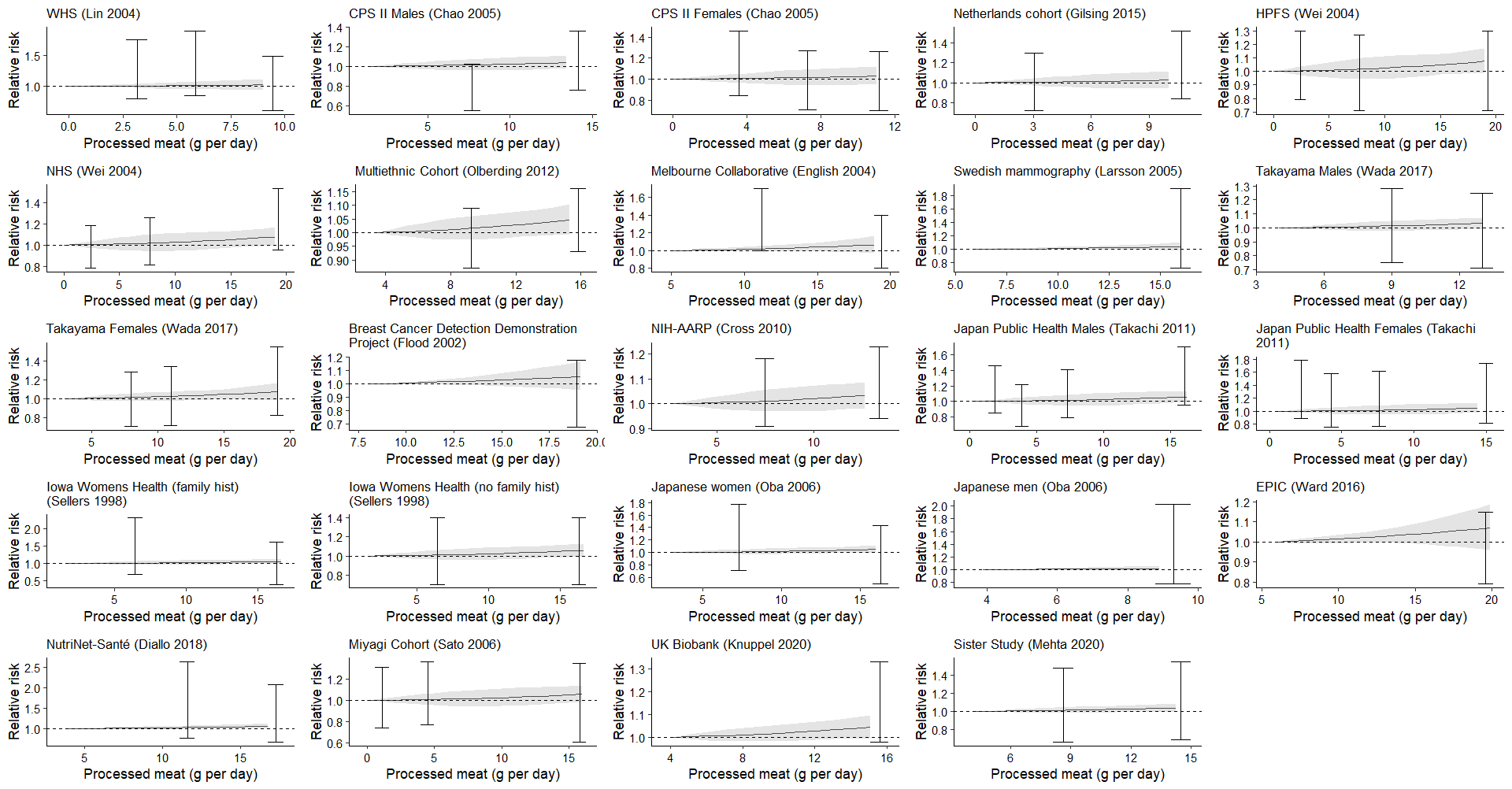


Supplemental Figure 7: Comparison of prediction intervals from the 2-stage restricted cubic spline DRMA fit to lower-consumer data for processed meat and colorectal cancer (<21 g/day) with the confidence intervals from the source studies’ consumption arms.


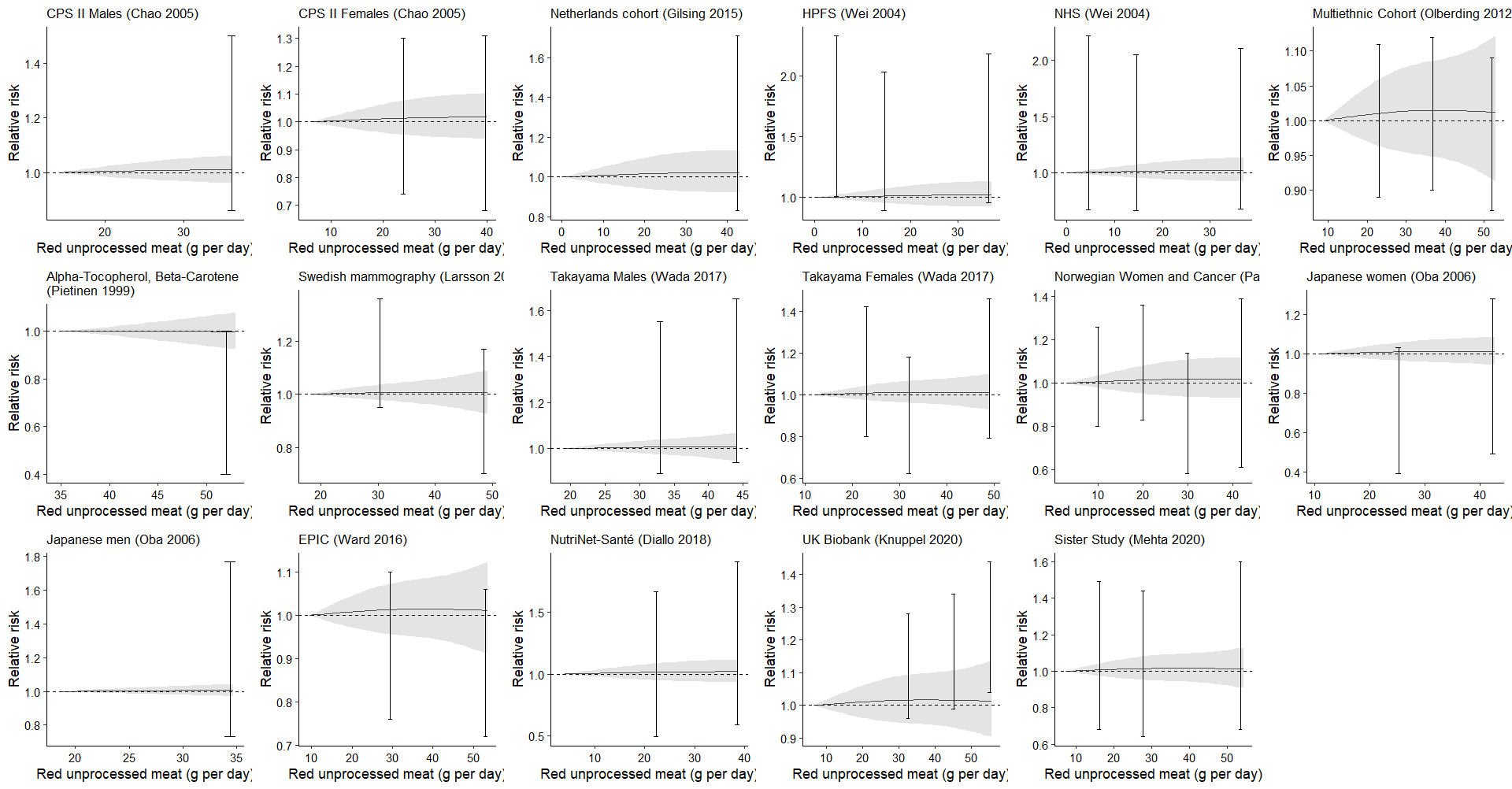


Supplemental Figure 8: Comparison of prediction intervals from the 2-stage restricted cubic spline DRMA fit to lower-consumer data for unprocessed red meat and colorectal cancer (<56 g/day) with the confidence intervals from the source studies’ consumption arms.

Supplemental Table 6: Relative risk estimated per gram for DRMA (mean and 95% Confidence interval) for models fit only to low-consumer data for processed meat consumption and colorectal cancer. Models are the empirical DR and the 2-stage RCS DR model.

| grams/day | Empirical LD | | 2-stage LD | |
| --- | --- | --- | --- | --- |
|  | Mean | 95% CI | Mean | 95% CI |
| 0 | 1.020 | (0.9264-1.1228) | 1 | (1-1) |
| 1 | 1.043 | (0.9904-1.0989) | 1.001 | (0.9884-1.0133) |
| 2 | 0.989 | (0.9595-1.0195) | 1.002 | (0.9772-1.0266) |
| 3 | 1.003 | (0.9608-1.0464) | 1.002 | (0.9667-1.0395) |
| 4 | 1.002 | (0.9832-1.0201) | 1.004 | (0.9573-1.052) |
| 5 | 1.010 | (0.9799-1.0414) | 1.005 | (0.9492-1.0637) |
| 6 | 1.014 | (0.975-1.0541) | 1.006 | (0.9425-1.0743) |
| 7 | 1.016 | (0.9723-1.0621) | 1.008 | (0.9377-1.0837) |
| 8 | 1.018 | (0.971-1.0672) | 1.010 | (0.9349-1.0916) |
| 9 | 1.019 | (0.9706-1.0701) | 1.013 | (0.9342-1.0979) |
| 10 | 1.021 | (0.9721-1.0732) | 1.016 | (0.9358-1.1025) |
| 11 | 1.027 | (0.9772-1.0799) | 1.019 | (0.9394-1.1057) |
| 12 | 1.033 | (0.9819-1.0858) | 1.023 | (0.9444-1.108) |
| 13 | 1.037 | (0.9847-1.0919) | 1.027 | (0.9502-1.11) |
| 14 | 1.038 | (0.9843-1.0951) | 1.031 | (0.9563-1.1124) |
| 15 | 1.039 | (0.9793-1.1015) | 1.036 | (0.9619-1.1159) |
| 16 | 1.038 | (0.9753-1.1043) | 1.041 | (0.9663-1.1212) |
| 17 | 1.039 | (0.9721-1.1111) | 1.046 | (0.969-1.1287) |
| 18 | 1.042 | (0.9431-1.1522) | 1.051 | (0.9698-1.1387) |
| 19 | 1.005 | (0.888-1.1381) | 1.056 | (0.9688-1.151) |
| 20 | 1.014 | (0.8678-1.1842) | 1.061 | (0.9663-1.1653) |

Supplemental Table 7: Relative risk estimated per gram for DRMA (mean and 95% Confidence interval) for models fit to the full range of consumption data for processed meat consumption and colorectal cancer. Models are the empirical DR and the 2-stage RCS DR model. GBD model is the reported RR from the GBD DRMA for 25, 50, 75, and 10o g/day, with linear extrapolation between these points for the purposes of comparison.

| grams/ day | Empirical All | | 2-stage All | | GBD | |
| --- | --- | --- | --- | --- | --- | --- |
|  | Mean | 95% CI | Mean | 95% CI | Mean | 95% CI |
| 0 | 1.003 | (0.9768-1.0298) | 1.000 | (1-1) | 1 | (1-1) |
| 1 | 1.003 | (0.9768-1.0298) | 1.005 | (1.0023-1.0081) | 1.002 | (1.0008-1.004) |
| 2 | 0.988 | (0.9572-1.0197) | 1.010 | (1.0046-1.0163) | 1.005 | (1.0016-1.008) |
| 3 | 0.995 | (0.9637-1.028) | 1.016 | (1.0069-1.0246) | 1.007 | (1.0024-1.012) |
| 4 | 1.002 | (0.9902-1.0138) | 1.021 | (1.0092-1.0328) | 1.010 | (1.0032-1.016) |
| 5 | 1.010 | (0.9951-1.0242) | 1.026 | (1.0115-1.0411) | 1.012 | (1.004-1.02) |
| 6 | 1.022 | (1.0001-1.0453) | 1.031 | (1.0139-1.0494) | 1.014 | (1.0048-1.024) |
| 7 | 1.006 | (0.9858-1.0267) | 1.037 | (1.0162-1.0577) | 1.017 | (1.0056-1.028) |
| 8 | 0.999 | (0.9893-1.0083) | 1.042 | (1.0186-1.066) | 1.019 | (1.0064-1.032) |
| 9 | 0.993 | (0.979-1.0072) | 1.047 | (1.021-1.0742) | 1.022 | (1.0072-1.036) |
| 10 | 1.000 | (0.9872-1.0135) | 1.052 | (1.0233-1.0824) | 1.024 | (1.008-1.04) |
| 11 | 1.001 | (0.9919-1.0111) | 1.058 | (1.0258-1.0905) | 1.026 | (1.0088-1.044) |
| 12 | 1.003 | (0.9865-1.0204) | 1.063 | (1.0282-1.0985) | 1.029 | (1.0096-1.048) |
| 13 | 1.006 | (0.9838-1.0294) | 1.068 | (1.0307-1.1064) | 1.031 | (1.0104-1.052) |
| 14 | 1.011 | (0.9833-1.0385) | 1.073 | (1.0331-1.1142) | 1.034 | (1.0112-1.056) |
| 15 | 1.016 | (0.9848-1.0478) | 1.078 | (1.0356-1.1219) | 1.036 | (1.012-1.06) |
| 16 | 1.022 | (0.9881-1.0572) | 1.083 | (1.0382-1.1294) | 1.038 | (1.0128-1.064) |
| 17 | 1.029 | (0.9921-1.0679) | 1.088 | (1.0407-1.1367) | 1.041 | (1.0136-1.068) |
| 18 | 1.040 | (1.0001-1.0809) | 1.092 | (1.0433-1.1439) | 1.043 | (1.0144-1.072) |
| 19 | 1.060 | (1.0119-1.11) | 1.097 | (1.0459-1.1509) | 1.046 | (1.0152-1.076) |
| 20 | 1.068 | (1.0175-1.1215) | 1.102 | (1.0485-1.1576) | 1.048 | (1.016-1.08) |
| 21 | 1.077 | (1.0235-1.1328) | 1.106 | (1.0512-1.1641) | 1.050 | (1.0168-1.084) |
| 22 | 1.077 | (1.0238-1.1321) | 1.111 | (1.0539-1.1704) | 1.053 | (1.0176-1.088) |
| 23 | 1.076 | (1.0198-1.1345) | 1.115 | (1.0566-1.1764) | 1.055 | (1.0184-1.092) |
| 24 | 1.084 | (1.027-1.145) | 1.119 | (1.0593-1.1822) | 1.058 | (1.0192-1.096) |
| 25 | 1.091 | (1.0324-1.1522) | 1.123 | (1.062-1.1878) | 1.060 | (1.02-1.1) |
| 26 | 1.097 | (1.0381-1.1597) | 1.127 | (1.0648-1.1931) | 1.061 | (1.0204-1.1012) |
| 27 | 1.085 | (1.0302-1.1432) | 1.131 | (1.0676-1.1983) | 1.062 | (1.0208-1.1024) |
| 28 | 1.094 | (1.0414-1.1495) | 1.135 | (1.0704-1.2032) | 1.062 | (1.0212-1.1036) |
| 29 | 1.104 | (1.0526-1.1571) | 1.139 | (1.0731-1.2079) | 1.063 | (1.0216-1.1048) |
| 30 | 1.109 | (1.0583-1.1628) | 1.142 | (1.0759-1.2125) | 1.064 | (1.022-1.106) |
| 31 | 1.103 | (1.0506-1.159) | 1.146 | (1.0787-1.2168) | 1.065 | (1.0224-1.1072) |
| 32 | 1.110 | (1.054-1.1698) | 1.149 | (1.0815-1.221) | 1.066 | (1.0228-1.1084) |
| 33 | 1.116 | (1.0573-1.1781) | 1.153 | (1.0843-1.225) | 1.066 | (1.0232-1.1096) |
| 34 | 1.121 | (1.0604-1.1844) | 1.156 | (1.0871-1.2289) | 1.067 | (1.0236-1.1108) |
| 35 | 1.111 | (1.0548-1.1708) | 1.159 | (1.0898-1.2326) | 1.068 | (1.024-1.112) |
| 36 | 1.114 | (1.0573-1.1729) | 1.162 | (1.0926-1.2362) | 1.069 | (1.0244-1.1132) |
| 37 | 1.116 | (1.0598-1.1746) | 1.165 | (1.0953-1.2396) | 1.070 | (1.0248-1.1144) |
| 38 | 1.118 | (1.0615-1.1777) | 1.168 | (1.0979-1.2429) | 1.070 | (1.0252-1.1156) |
| 39 | 1.120 | (1.0627-1.1814) | 1.171 | (1.1006-1.2461) | 1.071 | (1.0256-1.1168) |
| 40 | 1.120 | (1.0605-1.1825) | 1.174 | (1.1032-1.2493) | 1.072 | (1.026-1.118) |
| 41 | 1.122 | (1.0613-1.1863) | 1.177 | (1.1058-1.2523) | 1.073 | (1.0264-1.1192) |
| 42 | 1.124 | (1.0613-1.191) | 1.179 | (1.1083-1.2552) | 1.074 | (1.0268-1.1204) |
| 43 | 1.144 | (1.0688-1.2249) | 1.182 | (1.1107-1.2581) | 1.074 | (1.0272-1.1216) |
| 44 | 1.145 | (1.0688-1.2267) | 1.185 | (1.1131-1.2609) | 1.075 | (1.0276-1.1228) |
| 45 | 1.146 | (1.0687-1.2285) | 1.187 | (1.1155-1.2637) | 1.076 | (1.028-1.124) |
| 46 | 1.144 | (1.0488-1.2474) | 1.190 | (1.1177-1.2665) | 1.077 | (1.0284-1.1252) |
| 47 | 1.146 | (1.0522-1.2479) | 1.192 | (1.1199-1.2692) | 1.078 | (1.0288-1.1264) |
| 48 | 1.148 | (1.0557-1.2482) | 1.195 | (1.122-1.272) | 1.078 | (1.0292-1.1276) |
| 49 | 1.150 | (1.0595-1.2483) | 1.197 | (1.1241-1.2747) | 1.079 | (1.0296-1.1288) |
| 50 | 1.152 | (1.0613-1.2499) | 1.199 | (1.126-1.2774) | 1.080 | (1.03-1.13) |
| 51 | 1.154 | (1.063-1.2517) | 1.202 | (1.1279-1.2802) | 1.080 | (1.03-1.132) |
| 52 | 1.155 | (1.0648-1.2538) | 1.204 | (1.1296-1.283) | 1.081 | (1.03-1.134) |
| 53 | 1.158 | (1.0667-1.2562) | 1.206 | (1.1313-1.2859) | 1.081 | (1.03-1.136) |
| 54 | 1.174 | (1.0774-1.28) | 1.208 | (1.1328-1.2888) | 1.082 | (1.03-1.138) |
| 55 | 1.175 | (1.0783-1.2812) | 1.210 | (1.1343-1.2917) | 1.082 | (1.03-1.14) |
| 56 | 1.177 | (1.0794-1.2828) | 1.213 | (1.1356-1.2948) | 1.082 | (1.03-1.142) |
| 57 | 1.178 | (1.0806-1.2849) | 1.215 | (1.1369-1.2978) | 1.083 | (1.03-1.144) |
| 58 | 1.180 | (1.082-1.2874) | 1.217 | (1.1381-1.301) | 1.083 | (1.03-1.146) |
| 59 | 1.182 | (1.0835-1.2905) | 1.219 | (1.1391-1.3043) | 1.084 | (1.03-1.148) |
| 60 | 1.185 | (1.0852-1.294) | 1.221 | (1.1401-1.3076) | 1.084 | (1.03-1.15) |
| 61 | 1.188 | (1.087-1.298) | 1.223 | (1.141-1.311) | 1.084 | (1.03-1.152) |
| 62 | 1.191 | (1.0888-1.3023) | 1.225 | (1.1418-1.3145) | 1.085 | (1.03-1.154) |
| 63 | 1.194 | (1.0905-1.3069) | 1.227 | (1.1425-1.3181) | 1.085 | (1.03-1.156) |
| 64 | 1.197 | (1.092-1.3115) | 1.229 | (1.1432-1.3218) | 1.086 | (1.03-1.158) |
| 65 | 1.200 | (1.0934-1.3162) | 1.231 | (1.1437-1.3256) | 1.086 | (1.03-1.16) |
| 66 | 1.220 | (1.0956-1.3591) | 1.233 | (1.1443-1.3295) | 1.086 | (1.03-1.162) |
| 67 | 1.223 | (1.0967-1.3634) | 1.235 | (1.1447-1.3334) | 1.087 | (1.03-1.164) |
| 68 | 1.258 | (1.0997-1.4392) | 1.238 | (1.1451-1.3375) | 1.087 | (1.03-1.166) |
| 69 | 1.257 | (1.0834-1.4596) | 1.240 | (1.1454-1.3416) | 1.088 | (1.03-1.168) |
| 70 | 1.257 | (1.0693-1.4785) | 1.242 | (1.1457-1.3458) | 1.088 | (1.03-1.17) |
| 71 | 1.258 | (1.0567-1.4966) | 1.244 | (1.1459-1.35) | 1.088 | (1.03-1.172) |
| 72 | 1.258 | (1.0451-1.5144) | 1.246 | (1.1461-1.3544) | 1.089 | (1.03-1.174) |
| 73 | 1.177 | (1.0031-1.3805) | 1.248 | (1.1462-1.3588) | 1.089 | (1.03-1.176) |
| 74 | 1.173 | (0.9974-1.3794) | 1.250 | (1.1463-1.3633) | 1.090 | (1.03-1.178) |
| 75 | 1.169 | (0.9921-1.3783) | 1.252 | (1.1464-1.3678) | 1.090 | (1.03-1.18) |
| 76 | 1.166 | (0.9872-1.3772) | 1.254 | (1.1464-1.3724) | 1.090 | (1.03-1.1816) |
| 77 | 1.163 | (0.9826-1.3761) | 1.256 | (1.1464-1.377) | 1.091 | (1.03-1.1832) |
| 78 | 1.160 | (0.9785-1.3749) | 1.259 | (1.1463-1.3818) | 1.091 | (1.03-1.1848) |
| 79 | 1.157 | (0.9746-1.3737) | 1.261 | (1.1462-1.3865) | 1.092 | (1.03-1.1864) |
| 80 | 1.154 | (0.9711-1.3725) | 1.263 | (1.1461-1.3914) | 1.092 | (1.03-1.188) |
| 81 | 1.152 | (0.9678-1.3713) | 1.265 | (1.1459-1.3962) | 1.092 | (1.03-1.1896) |
| 82 | 1.150 | (0.9649-1.3701) | 1.267 | (1.1457-1.4012) | 1.093 | (1.03-1.1912) |
| 83 | 1.148 | (0.9621-1.3689) | 1.269 | (1.1455-1.4061) | 1.093 | (1.03-1.1928) |
| 84 | 1.146 | (0.9596-1.3678) | 1.271 | (1.1453-1.4112) | 1.094 | (1.03-1.1944) |
| 85 | 1.144 | (0.9574-1.3666) | 1.273 | (1.145-1.4162) | 1.094 | (1.03-1.196) |
| 86 | 1.142 | (0.9553-1.3654) | 1.276 | (1.1447-1.4214) | 1.094 | (1.03-1.1976) |
| 87 | 1.141 | (0.9535-1.3643) | 1.278 | (1.1444-1.4265) | 1.095 | (1.03-1.1992) |
| 88 | 1.139 | (0.9519-1.3633) | 1.280 | (1.1441-1.4317) | 1.095 | (1.03-1.2008) |
| 89 | 1.138 | (0.9504-1.3622) | 1.282 | (1.1438-1.437) | 1.096 | (1.03-1.2024) |
| 90 | 1.137 | (0.9492-1.3613) | 1.284 | (1.1434-1.4423) | 1.096 | (1.03-1.204) |
| 91 | 1.136 | (0.9481-1.3604) | 1.286 | (1.143-1.4476) | 1.096 | (1.03-1.2056) |
| 92 | 1.135 | (0.9472-1.3595) | 1.289 | (1.1427-1.453) | 1.097 | (1.03-1.2072) |
| 93 | 1.020 | (0.8381-1.2413) | 1.291 | (1.1423-1.4584) | 1.097 | (1.03-1.2088) |
| 94 | 1.020 | (0.8386-1.2397) | 1.293 | (1.1418-1.4638) | 1.098 | (1.03-1.2104) |
| 95 | 1.019 | (0.839-1.2385) | 1.295 | (1.1414-1.4693) | 1.098 | (1.03-1.212) |
| 96 | 1.019 | (0.8394-1.2375) | 1.297 | (1.141-1.4748) | 1.098 | (1.03-1.2136) |
| 97 | 1.019 | (0.8397-1.2369) | 1.299 | (1.1405-1.4804) | 1.099 | (1.03-1.2152) |
| 98 | 1.019 | (0.8399-1.2366) | 1.302 | (1.14-1.486) | 1.099 | (1.03-1.2168) |
| 99 | 1.019 | (0.84-1.2366) | 1.304 | (1.1395-1.4917) | 1.100 | (1.03-1.2184) |
| 100 | 1.019 | (0.8401-1.237) | 1.306 | (1.139-1.4973) |  |  |
| 101 | 1.028 | (0.6471-1.6327) | 1.308 | (1.1385-1.503) |  |  |
| 102 | 1.034 | (0.6533-1.6358) | 1.310 | (1.138-1.5088) |  |  |
| 103 | 1.040 | (0.6597-1.6397) | 1.313 | (1.1375-1.5146) |  |  |
| 104 | 1.047 | (0.6661-1.6446) | 1.315 | (1.137-1.5204) |  |  |
| 105 | 1.054 | (0.6726-1.6504) | 1.317 | (1.1364-1.5262) |  |  |
| 106 | 1.061 | (0.679-1.6572) | 1.319 | (1.1359-1.5321) |  |  |
| 107 | 1.068 | (0.6854-1.665) | 1.321 | (1.1353-1.538) |  |  |
| 108 | 1.076 | (0.6917-1.674) | 1.324 | (1.1348-1.5439) |  |  |
| 109 | 1.084 | (0.6978-1.684) | 1.326 | (1.1342-1.5499) |  |  |
| 110 | 1.092 | (0.7037-1.6953) | 1.328 | (1.1336-1.5559) |  |  |
| 111 | 1.101 | (0.7094-1.7077) | 1.330 | (1.133-1.562) |  |  |
| 112 | 1.109 | (0.7148-1.7213) | 1.333 | (1.1324-1.5681) |  |  |
| 113 | 1.118 | (0.7199-1.7361) | 1.335 | (1.1319-1.5742) |  |  |
| 114 | 1.127 | (0.7246-1.7521) | 1.337 | (1.1313-1.5803) |  |  |
| 115 | 1.136 | (0.7289-1.7693) | 1.339 | (1.1306-1.5865) |  |  |
| 116 | 1.145 | (0.7329-1.7878) | 1.342 | (1.13-1.5927) |  |  |
| 117 | 1.154 | (0.7364-1.8074) | 1.344 | (1.1294-1.5989) |  |  |
| 118 | 1.163 | (0.7395-1.8283) | 1.346 | (1.1288-1.6052) |  |  |
| 119 | 1.172 | (0.7423-1.8504) | 1.348 | (1.1282-1.6115) |  |  |
| 120 | 1.181 | (0.7447-1.8737) | 1.351 | (1.1275-1.6178) |  |  |
| 121 | 1.191 | (0.7467-1.8984) | 1.353 | (1.1269-1.6242) |  |  |
| 122 | 1.200 | (0.7483-1.9243) | 1.355 | (1.1263-1.6306) |  |  |

Supplemental Table 8: Relative risk estimated per gram for DRMA (mean and 95% Confidence interval) for models fit only to low-consumer data for unprocessed red meat consumption and colorectal cancer. Models are the empirical DR and the 2-stage RCS DR model.

| grams/day | Empirical DR LD | | 2-stage RCS LD | |
| --- | --- | --- | --- | --- |
|  | Mean | 95% CI | Mean | 95% CI |
| 0 | 1.013 | (0.8391-1.2235) | 1 | (1-1) |
| 1 | 1.074 | (0.997-1.1579) | 1.001 | (0.9966-1.0048) |
| 2 | 1.087 | (0.9609-1.2292) | 1.001 | (0.9932-1.0096) |
| 3 | 1.082 | (0.9243-1.2659) | 1.002 | (0.9898-1.0144) |
| 4 | 1.032 | (0.9467-1.1255) | 1.003 | (0.9864-1.0193) |
| 5 | 1.002 | (0.9649-1.0401) | 1.003 | (0.9831-1.0242) |
| 6 | 1.010 | (0.9486-1.0763) | 1.004 | (0.9798-1.029) |
| 7 | 1.020 | (0.94-1.1069) | 1.005 | (0.9765-1.0339) |
| 8 | 1.029 | (0.9361-1.1312) | 1.005 | (0.9732-1.0388) |
| 9 | 1.037 | (0.9348-1.1497) | 1.006 | (0.97-1.0436) |
| 10 | 0.999 | (0.9883-1.0104) | 1.007 | (0.9669-1.0484) |
| 11 | 0.996 | (0.9852-1.0067) | 1.007 | (0.9638-1.0531) |
| 12 | 0.988 | (0.9712-1.0059) | 1.008 | (0.9608-1.0578) |
| 13 | 0.995 | (0.9747-1.0163) | 1.009 | (0.9578-1.0624) |
| 14 | 0.992 | (0.9624-1.0218) | 1.009 | (0.955-1.0669) |
| 15 | 0.990 | (0.9536-1.0272) | 1.010 | (0.9522-1.0713) |
| 16 | 0.988 | (0.9465-1.0322) | 1.011 | (0.9495-1.0757) |
| 17 | 0.988 | (0.9406-1.0369) | 1.011 | (0.9469-1.0799) |
| 18 | 0.987 | (0.9356-1.0413) | 1.012 | (0.9445-1.0839) |
| 19 | 1.012 | (0.9941-1.0292) | 1.012 | (0.9421-1.0879) |
| 20 | 1.015 | (0.994-1.036) | 1.013 | (0.9398-1.0916) |
| 21 | 1.021 | (0.9881-1.0554) | 1.013 | (0.9377-1.0953) |
| 22 | 1.023 | (0.9813-1.0669) | 1.014 | (0.9357-1.0987) |
| 23 | 1.023 | (0.9749-1.0734) | 1.014 | (0.9338-1.102) |
| 24 | 1.022 | (0.9691-1.0769) | 1.015 | (0.9321-1.1051) |
| 25 | 1.019 | (0.9638-1.0781) | 1.015 | (0.9305-1.108) |
| 26 | 1.017 | (0.9593-1.0779) | 1.016 | (0.929-1.1107) |
| 27 | 1.014 | (0.9554-1.0768) | 1.016 | (0.9276-1.1132) |
| 28 | 1.012 | (0.9521-1.0754) | 1.017 | (0.9264-1.1154) |
| 29 | 1.010 | (0.9495-1.0742) | 1.017 | (0.9254-1.1175) |
| 30 | 1.008 | (0.9474-1.0732) | 1.017 | (0.9244-1.1193) |
| 31 | 1.007 | (0.9458-1.0727) | 1.018 | (0.9236-1.1209) |
| 32 | 1.006 | (0.9444-1.0725) | 1.018 | (0.923-1.1223) |
| 33 | 1.006 | (0.9433-1.0724) | 1.018 | (0.9224-1.1235) |
| 34 | 1.005 | (0.9421-1.0723) | 1.018 | (0.922-1.1245) |
| 35 | 1.004 | (0.941-1.0722) | 1.018 | (0.9216-1.1253) |
| 36 | 1.004 | (0.9399-1.0719) | 1.019 | (0.9213-1.126) |
| 37 | 1.003 | (0.9388-1.0713) | 1.019 | (0.921-1.1265) |
| 38 | 0.987 | (0.922-1.0567) | 1.019 | (0.9208-1.127) |
| 39 | 0.986 | (0.9216-1.0559) | 1.019 | (0.9205-1.1275) |
| 40 | 0.975 | (0.909-1.0465) | 1.019 | (0.9202-1.128) |
| 41 | 0.974 | (0.9084-1.045) | 1.019 | (0.9199-1.1285) |
| 42 | 0.973 | (0.9075-1.0432) | 1.019 | (0.9194-1.129) |
| 43 | 0.971 | (0.9039-1.043) | 1.019 | (0.9189-1.1296) |
| 44 | 0.976 | (0.9074-1.0501) | 1.019 | (0.9183-1.1304) |
| 45 | 0.973 | (0.9051-1.0469) | 1.019 | (0.9175-1.1312) |
| 46 | 0.945 | (0.8725-1.0235) | 1.019 | (0.9166-1.1322) |
| 47 | 0.943 | (0.8694-1.0223) | 1.019 | (0.9155-1.1334) |
| 48 | 0.940 | (0.8652-1.0214) | 1.019 | (0.9143-1.1348) |
| 49 | 0.937 | (0.86-1.0207) | 1.018 | (0.9129-1.1363) |
| 50 | 0.933 | (0.8538-1.0201) | 1.018 | (0.9113-1.1381) |
| 51 | 0.886 | (0.7494-1.0477) | 1.018 | (0.9096-1.14) |
| 52 | 0.885 | (0.7499-1.0448) | 1.018 | (0.9077-1.1422) |
| 53 | 0.884 | (0.7501-1.0421) | 1.018 | (0.9057-1.1446) |
| 54 | 0.767 | (0.5431-1.0844) | 1.018 | (0.9035-1.1471) |
| 55 | 0.6 | (0.3795-0.9487) | 1.018 | (0.9012-1.1498) |

Supplemental Table 9: Relative risk estimated per gram for DRMA (mean and 95% Confidence interval) for models fit to the full range of consumption data for unprocessed red meat consumption and colorectal cancer. Models are the empirical DR and the 2-stage RCS DR model. GBD model is the reported RR from the GBD DRMA for, 50, 100, and 150 g/day, with linear extrapolation between for comparison.

| grams/day | Empirical DR | | 2-stage RCS All | | GBD | |
| --- | --- | --- | --- | --- | --- | --- |
|  | Mean | 95% CI | Mean | 95% CI | Mean | 95% CI |
| 0 | 1.057 | (0.9988-1.1188) | 1.000 | (1-1) | 1.000 | (1-1) |
| 1 | 1.057 | (0.9988-1.1188) | 1.002 | (1-1.0039) | 1.002 | (1.0004-1.0032) |
| 2 | 1.081 | (0.9970-1.1727) | 1.004 | (0.9999-1.0077) | 1.003 | (1.0008-1.0064) |
| 3 | 1.094 | (0.9961-1.2014) | 1.006 | (0.9999-1.0116) | 1.005 | (1.0012-1.0096) |
| 4 | 1.102 | (0.9947-1.2201) | 1.008 | (0.9998-1.0155) | 1.006 | (1.0016-1.0128) |
| 5 | 1.106 | (0.9934-1.2323) | 1.010 | (0.9998-1.0194) | 1.008 | (1.002-1.016) |
| 6 | 1.097 | (0.9871-1.2201) | 1.011 | (0.9998-1.0234) | 1.010 | (1.0024-1.0192) |
| 7 | 1.003 | (0.9943-1.0125) | 1.013 | (0.9997-1.0273) | 1.011 | (1.0028-1.0224) |
| 8 | 1.008 | (0.9898-1.0260) | 1.015 | (0.9997-1.0313) | 1.013 | (1.0032-1.0256) |
| 9 | 1.013 | (0.9865-1.0402) | 1.017 | (0.9996-1.0352) | 1.014 | (1.0036-1.0288) |
| 10 | 0.998 | (0.9928-1.0026) | 1.019 | (0.9996-1.0392) | 1.016 | (1.004-1.032) |
| 11 | 0.996 | (0.9864-1.0061) | 1.021 | (0.9996-1.0432) | 1.018 | (1.0044-1.0352) |
| 12 | 0.992 | (0.9786-1.0052) | 1.023 | (0.9995-1.0472) | 1.019 | (1.0048-1.0384) |
| 13 | 0.997 | (0.9786-1.0155) | 1.025 | (0.9995-1.0512) | 1.021 | (1.0052-1.0416) |
| 14 | 0.996 | (0.9723-1.0206) | 1.027 | (0.9995-1.0552) | 1.022 | (1.0056-1.0448) |
| 15 | 1.003 | (0.9897-1.0155) | 1.029 | (0.9995-1.0592) | 1.024 | (1.006-1.048) |
| 16 | 1.003 | (0.9812-1.0254) | 1.031 | (0.9994-1.0631) | 1.026 | (1.0064-1.0512) |
| 17 | 1.004 | (0.9764-1.0330) | 1.033 | (0.9994-1.0671) | 1.027 | (1.0068-1.0544) |
| 18 | 1.006 | (0.9728-1.0399) | 1.035 | (0.9994-1.0711) | 1.029 | (1.0072-1.0576) |
| 19 | 1.013 | (0.9969-1.0297) | 1.037 | (0.9994-1.0751) | 1.030 | (1.0076-1.0608) |
| 20 | 1.015 | (0.9976-1.0321) | 1.038 | (0.9994-1.0791) | 1.032 | (1.008-1.064) |
| 21 | 1.010 | (0.9914-1.0284) | 1.040 | (0.9994-1.083) | 1.034 | (1.0084-1.0672) |
| 22 | 1.003 | (0.9963-1.0096) | 1.042 | (0.9994-1.087) | 1.035 | (1.0088-1.0704) |
| 23 | 1.005 | (0.9943-1.0162) | 1.044 | (0.9994-1.0909) | 1.037 | (1.0092-1.0736) |
| 24 | 1.008 | (0.9925-1.0228) | 1.046 | (0.9994-1.0948) | 1.038 | (1.0096-1.0768) |
| 25 | 1.008 | (0.9949-1.0217) | 1.048 | (0.9995-1.0987) | 1.040 | (1.01-1.08) |
| 26 | 1.012 | (0.9931-1.0308) | 1.050 | (0.9995-1.1026) | 1.042 | (1.0104-1.0832) |
| 27 | 1.014 | (0.9915-1.0372) | 1.052 | (0.9995-1.1064) | 1.043 | (1.0108-1.0864) |
| 28 | 1.016 | (0.9901-1.0422) | 1.053 | (0.9996-1.1103) | 1.045 | (1.0112-1.0896) |
| 29 | 1.017 | (0.9889-1.0467) | 1.055 | (0.9996-1.1141) | 1.046 | (1.0116-1.0928) |
| 30 | 1.019 | (0.9877-1.0509) | 1.057 | (0.9997-1.1179) | 1.048 | (1.012-1.096) |
| 31 | 1.010 | (1.0001-1.0201) | 1.059 | (0.9998-1.1216) | 1.050 | (1.0124-1.0992) |
| 32 | 1.019 | (1.0026-1.0355) | 1.061 | (0.9998-1.1254) | 1.051 | (1.0128-1.1024) |
| 33 | 1.026 | (1.0035-1.0484) | 1.063 | (0.9999-1.1291) | 1.053 | (1.0132-1.1056) |
| 34 | 1.031 | (1.0037-1.0581) | 1.064 | (1-1.1327) | 1.054 | (1.0136-1.1088) |
| 35 | 1.034 | (1.0034-1.0652) | 1.066 | (1.0001-1.1364) | 1.056 | (1.014-1.112) |
| 36 | 1.019 | (0.9802-1.0591) | 1.068 | (1.0002-1.14) | 1.058 | (1.0144-1.1152) |
| 37 | 1.020 | (0.9763-1.0664) | 1.070 | (1.0003-1.1435) | 1.059 | (1.0148-1.1184) |
| 38 | 1.022 | (0.9755-1.0712) | 1.071 | (1.0004-1.147) | 1.061 | (1.0152-1.1216) |
| 39 | 1.025 | (0.9766-1.0748) | 1.073 | (1.0006-1.1505) | 1.062 | (1.0156-1.1248) |
| 40 | 1.027 | (0.9785-1.0778) | 1.075 | (1.0007-1.1539) | 1.064 | (1.016-1.128) |
| 41 | 1.029 | (0.9801-1.0806) | 1.076 | (1.0009-1.1573) | 1.066 | (1.0164-1.1312) |
| 42 | 1.031 | (0.9812-1.0834) | 1.078 | (1.001-1.1606) | 1.067 | (1.0168-1.1344) |
| 43 | 1.034 | (0.9807-1.0913) | 1.079 | (1.0012-1.1639) | 1.069 | (1.0172-1.1376) |
| 44 | 1.039 | (0.9836-1.0982) | 1.081 | (1.0014-1.1671) | 1.070 | (1.0176-1.1408) |
| 45 | 1.042 | (0.9843-1.1021) | 1.083 | (1.0016-1.1702) | 1.072 | (1.018-1.144) |
| 46 | 1.044 | (0.9853-1.1064) | 1.084 | (1.0018-1.1733) | 1.074 | (1.0184-1.1472) |
| 47 | 1.047 | (0.9866-1.1114) | 1.086 | (1.002-1.1764) | 1.075 | (1.0188-1.1504) |
| 48 | 1.050 | (0.9880-1.1167) | 1.087 | (1.0022-1.1794) | 1.077 | (1.0192-1.1536) |
| 49 | 1.041 | (0.9872-1.0968) | 1.089 | (1.0025-1.1823) | 1.078 | (1.0196-1.1568) |
| 50 | 1.044 | (0.9884-1.1035) | 1.090 | (1.0027-1.1851) | 1.080 | (1.02-1.16) |
| 51 | 1.046 | (0.9866-1.1097) | 1.092 | (1.003-1.1879) | 1.082 | (1.0212-1.1618) |
| 52 | 1.049 | (0.9872-1.1147) | 1.093 | (1.0033-1.1906) | 1.083 | (1.0224-1.1636) |
| 53 | 1.052 | (0.9879-1.1193) | 1.094 | (1.0035-1.1933) | 1.085 | (1.0236-1.1654) |
| 54 | 1.054 | (0.9886-1.1235) | 1.096 | (1.0038-1.1959) | 1.086 | (1.0248-1.1672) |
| 55 | 1.059 | (0.9895-1.1334) | 1.097 | (1.0041-1.1984) | 1.088 | (1.026-1.169) |
| 56 | 1.061 | (0.9904-1.1375) | 1.098 | (1.0044-1.2008) | 1.090 | (1.0272-1.1708) |
| 57 | 1.046 | (0.9773-1.1193) | 1.099 | (1.0048-1.2031) | 1.091 | (1.0284-1.1726) |
| 58 | 1.050 | (0.9790-1.1255) | 1.101 | (1.0051-1.2054) | 1.093 | (1.0296-1.1744) |
| 59 | 1.055 | (0.9807-1.1353) | 1.102 | (1.0054-1.2076) | 1.094 | (1.0308-1.1762) |
| 60 | 1.059 | (0.9832-1.1412) | 1.103 | (1.0058-1.2098) | 1.096 | (1.032-1.178) |
| 61 | 1.063 | (0.9860-1.1469) | 1.104 | (1.0061-1.2118) | 1.098 | (1.0332-1.1798) |
| 62 | 1.068 | (0.9890-1.1526) | 1.105 | (1.0065-1.2138) | 1.099 | (1.0344-1.1816) |
| 63 | 1.072 | (0.9921-1.1581) | 1.106 | (1.0069-1.2158) | 1.101 | (1.0356-1.1834) |
| 64 | 1.076 | (0.9953-1.1638) | 1.107 | (1.0072-1.2176) | 1.102 | (1.0368-1.1852) |
| 65 | 1.081 | (0.9985-1.1695) | 1.108 | (1.0076-1.2195) | 1.104 | (1.038-1.187) |
| 66 | 1.061 | (0.9845-1.1426) | 1.109 | (1.008-1.2212) | 1.106 | (1.0392-1.1888) |
| 67 | 1.066 | (0.9875-1.1502) | 1.110 | (1.0084-1.2229) | 1.107 | (1.0404-1.1906) |
| 68 | 1.071 | (0.9903-1.1576) | 1.111 | (1.0088-1.2245) | 1.109 | (1.0416-1.1924) |
| 69 | 1.054 | (0.9758-1.1391) | 1.112 | (1.0092-1.2261) | 1.110 | (1.0428-1.1942) |
| 70 | 1.057 | (0.9773-1.1422) | 1.113 | (1.0096-1.2276) | 1.112 | (1.044-1.196) |
| 71 | 1.059 | (0.9788-1.1453) | 1.114 | (1.0099-1.2291) | 1.114 | (1.0452-1.1978) |
| 72 | 1.061 | (0.9803-1.1483) | 1.115 | (1.0103-1.2305) | 1.115 | (1.0464-1.1996) |
| 73 | 1.063 | (0.9818-1.1512) | 1.116 | (1.0107-1.2318) | 1.117 | (1.0476-1.2014) |
| 74 | 1.065 | (0.9832-1.1539) | 1.117 | (1.0111-1.2331) | 1.118 | (1.0488-1.2032) |
| 75 | 1.067 | (0.9847-1.1566) | 1.117 | (1.0115-1.2344) | 1.120 | (1.05-1.205) |
| 76 | 1.069 | (0.9863-1.1592) | 1.118 | (1.0119-1.2356) | 1.122 | (1.0512-1.2068) |
| 77 | 1.071 | (0.9878-1.1617) | 1.119 | (1.0123-1.2368) | 1.123 | (1.0524-1.2086) |
| 78 | 1.073 | (0.9894-1.1642) | 1.120 | (1.0127-1.2379) | 1.125 | (1.0536-1.2104) |
| 79 | 1.075 | (0.9910-1.1665) | 1.120 | (1.013-1.239) | 1.126 | (1.0548-1.2122) |
| 80 | 1.077 | (0.9927-1.1689) | 1.121 | (1.0134-1.24) | 1.128 | (1.056-1.214) |
| 81 | 1.103 | (1.0071-1.2091) | 1.122 | (1.0138-1.2411) | 1.130 | (1.0572-1.2158) |
| 82 | 1.105 | (1.0089-1.2109) | 1.122 | (1.0141-1.242) | 1.131 | (1.0584-1.2176) |
| 83 | 1.107 | (1.0106-1.2127) | 1.123 | (1.0145-1.243) | 1.133 | (1.0596-1.2194) |
| 84 | 1.109 | (1.0124-1.2144) | 1.124 | (1.0148-1.2439) | 1.134 | (1.0608-1.2212) |
| 85 | 1.111 | (1.0142-1.2161) | 1.124 | (1.0151-1.2448) | 1.136 | (1.062-1.223) |
| 86 | 1.112 | (1.0159-1.2178) | 1.125 | (1.0154-1.2456) | 1.138 | (1.0632-1.2248) |
| 87 | 1.114 | (1.0177-1.2195) | 1.125 | (1.0157-1.2465) | 1.139 | (1.0644-1.2266) |
| 88 | 1.116 | (1.0194-1.2211) | 1.126 | (1.016-1.2473) | 1.141 | (1.0656-1.2284) |
| 89 | 1.117 | (1.0210-1.2228) | 1.126 | (1.0163-1.2481) | 1.142 | (1.0668-1.2302) |
| 90 | 1.119 | (1.0227-1.2246) | 1.127 | (1.0166-1.2488) | 1.144 | (1.068-1.232) |
| 91 | 1.152 | (1.0539-1.2582) | 1.127 | (1.0169-1.2496) | 1.146 | (1.0692-1.2338) |
| 92 | 1.152 | (1.0543-1.2594) | 1.128 | (1.0171-1.2503) | 1.147 | (1.0704-1.2356) |
| 93 | 1.153 | (1.0542-1.2612) | 1.128 | (1.0173-1.251) | 1.149 | (1.0716-1.2374) |
| 94 | 1.154 | (1.0539-1.2635) | 1.129 | (1.0175-1.2517) | 1.150 | (1.0728-1.2392) |
| 95 | 1.160 | (1.0544-1.2752) | 1.129 | (1.0177-1.2524) | 1.152 | (1.074-1.241) |
| 96 | 1.160 | (1.0541-1.2775) | 1.129 | (1.0179-1.2531) | 1.154 | (1.0752-1.2428) |
| 97 | 1.161 | (1.0539-1.2798) | 1.130 | (1.0181-1.2538) | 1.155 | (1.0764-1.2446) |
| 98 | 1.162 | (1.0539-1.2820) | 1.130 | (1.0182-1.2545) | 1.157 | (1.0776-1.2464) |
| 99 | 1.163 | (1.0539-1.2841) | 1.131 | (1.0183-1.2551) | 1.158 | (1.0788-1.2482) |
| 100 | 1.164 | (1.0541-1.2861) | 1.131 | (1.0184-1.2558) | 1.160 | (1.08-1.25) |
| 101 | 1.186 | (1.0737-1.3109) | 1.131 | (1.0185-1.2564) | 1.161 | (1.0804-1.252) |
| 102 | 1.187 | (1.0739-1.3126) | 1.132 | (1.0185-1.2571) | 1.162 | (1.0808-1.254) |
| 103 | 1.188 | (1.0739-1.3142) | 1.132 | (1.0186-1.2578) | 1.164 | (1.0812-1.256) |
| 104 | 1.207 | (1.0847-1.3425) | 1.132 | (1.0186-1.2584) | 1.165 | (1.0816-1.258) |
| 105 | 1.207 | (1.0843-1.3435) | 1.132 | (1.0186-1.2591) | 1.166 | (1.082-1.26) |
| 106 | 1.207 | (1.0838-1.3444) | 1.133 | (1.0186-1.2598) | 1.167 | (1.0824-1.262) |
| 107 | 1.207 | (1.0834-1.3453) | 1.133 | (1.0185-1.2605) | 1.168 | (1.0828-1.264) |
| 108 | 1.207 | (1.0829-1.3461) | 1.133 | (1.0185-1.2612) | 1.170 | (1.0832-1.266) |
| 109 | 1.207 | (1.0823-1.3469) | 1.134 | (1.0184-1.2619) | 1.171 | (1.0836-1.268) |
| 110 | 1.207 | (1.0818-1.3476) | 1.134 | (1.0182-1.2626) | 1.172 | (1.084-1.27) |
| 111 | 1.195 | (1.0691-1.3361) | 1.134 | (1.0181-1.2633) | 1.173 | (1.0844-1.272) |
| 112 | 1.195 | (1.0685-1.3366) | 1.134 | (1.0179-1.2641) | 1.174 | (1.0848-1.274) |
| 113 | 1.195 | (1.0678-1.3371) | 1.135 | (1.0178-1.2648) | 1.176 | (1.0852-1.276) |
| 114 | 1.195 | (1.0672-1.3375) | 1.135 | (1.0175-1.2656) | 1.177 | (1.0856-1.278) |
| 115 | 1.195 | (1.0665-1.3380) | 1.135 | (1.0173-1.2664) | 1.178 | (1.086-1.28) |
| 116 | 1.194 | (1.0658-1.3384) | 1.135 | (1.0171-1.2672) | 1.179 | (1.0864-1.282) |
| 117 | 1.194 | (1.0651-1.3388) | 1.135 | (1.0168-1.268) | 1.180 | (1.0868-1.284) |
| 118 | 1.194 | (1.0643-1.3391) | 1.136 | (1.0165-1.2689) | 1.182 | (1.0872-1.286) |
| 119 | 1.194 | (1.0634-1.3395) | 1.136 | (1.0161-1.2697) | 1.183 | (1.0876-1.288) |
| 120 | 1.185 | (1.0402-1.3505) | 1.136 | (1.0158-1.2706) | 1.184 | (1.088-1.29) |
| 121 | 1.184 | (1.0294-1.3627) | 1.136 | (1.0154-1.2715) | 1.185 | (1.0884-1.292) |
| 122 | 1.184 | (1.0281-1.3632) | 1.136 | (1.015-1.2725) | 1.186 | (1.0888-1.294) |
| 123 | 1.183 | (1.0268-1.3636) | 1.137 | (1.0146-1.2734) | 1.188 | (1.0892-1.296) |
| 124 | 1.183 | (1.0074-1.3900) | 1.137 | (1.0142-1.2744) | 1.189 | (1.0896-1.298) |
| 125 | 1.182 | (1.0057-1.3901) | 1.137 | (1.0137-1.2754) | 1.190 | (1.09-1.3) |
| 126 | 1.181 | (1.0039-1.3902) | 1.137 | (1.0133-1.2764) | 1.191 | (1.0904-1.302) |
| 127 | 1.180 | (1.0020-1.3903) | 1.137 | (1.0128-1.2775) | 1.192 | (1.0908-1.304) |
| 128 | 1.179 | (1.0001-1.3905) | 1.138 | (1.0123-1.2785) | 1.194 | (1.0912-1.306) |
| 129 | 1.178 | (0.9981-1.3907) | 1.138 | (1.0117-1.2796) | 1.195 | (1.0916-1.308) |
| 130 | 1.177 | (0.9960-1.3910) | 1.138 | (1.0112-1.2808) | 1.196 | (1.092-1.31) |
| 131 | 1.176 | (0.9938-1.3913) | 1.138 | (1.0106-1.2819) | 1.197 | (1.0924-1.312) |
| 132 | 1.175 | (0.9916-1.3916) | 1.138 | (1.0101-1.2831) | 1.198 | (1.0928-1.314) |
| 133 | 1.174 | (0.9894-1.3920) | 1.139 | (1.0095-1.2843) | 1.200 | (1.0932-1.316) |
| 134 | 1.169 | (0.9468-1.4424) | 1.139 | (1.0088-1.2855) | 1.201 | (1.0936-1.318) |
| 135 | 1.167 | (0.9438-1.4430) | 1.139 | (1.0082-1.2867) | 1.202 | (1.094-1.32) |
| 136 | 1.165 | (0.9409-1.4436) | 1.139 | (1.0076-1.288) | 1.203 | (1.0944-1.322) |
| 137 | 1.164 | (0.9378-1.4443) | 1.139 | (1.0069-1.2893) | 1.204 | (1.0948-1.324) |
| 138 | 1.167 | (0.8649-1.5754) | 1.140 | (1.0062-1.2906) | 1.206 | (1.0952-1.326) |
| 139 | 1.166 | (0.8627-1.5767) | 1.140 | (1.0055-1.2919) | 1.207 | (1.0956-1.328) |
| 140 | 1.165 | (0.8605-1.5779) | 1.140 | (1.0048-1.2932) | 1.208 | (1.096-1.33) |
| 141 | 1.164 | (0.8583-1.5791) | 1.140 | (1.0041-1.2946) | 1.209 | (1.0964-1.332) |
| 142 | 1.163 | (0.8560-1.5802) | 1.140 | (1.0034-1.296) | 1.210 | (1.0968-1.334) |
| 143 | 1.162 | (0.8537-1.5812) | 1.141 | (1.0026-1.2974) | 1.212 | (1.0972-1.336) |
| 144 | 1.161 | (0.8513-1.5821) | 1.141 | (1.0019-1.2988) | 1.213 | (1.0976-1.338) |
| 145 | 1.109 | (0.6845-1.7983) | 1.141 | (1.0011-1.3003) | 1.214 | (1.098-1.34) |
| 146 | 1.107 | (0.6825-1.7959) | 1.141 | (1.0003-1.3017) | 1.215 | (1.0984-1.342) |
| 147 | 1.105 | (0.6805-1.7933) | 1.141 | (0.9995-1.3032) | 1.216 | (1.0988-1.344) |
| 148 | 1.102 | (0.6784-1.7905) | 1.142 | (0.9987-1.3047) | 1.218 | (1.0992-1.346) |
| 149 | 1.100 | (0.6764-1.7877) | 1.142 | (0.9979-1.3062) | 1.219 | (1.0996-1.348) |
| 150 | 1.097 | (0.6743-1.7848) | 1.142 | (0.9971-1.3077) | 1.220 | (1.1-1.35) |
| 151 | 1.094 | (0.6722-1.7818) | 1.142 | (0.9962-1.3093) | 1.221 | (1.1002-1.3522) |
| 152 | 1.092 | (0.6701-1.7787) | 1.142 | (0.9954-1.3108) | 1.222 | (1.1004-1.3544) |
| 153 | 1.408 | (1.0329-1.9185) | 1.142 | (0.9945-1.3124) | 1.223 | (1.1006-1.3566) |
| 154 | 1.405 | (1.0282-1.9194) | 1.143 | (0.9937-1.314) | 1.224 | (1.1008-1.3588) |
| 155 | 1.402 | (1.0232-1.9208) | 1.143 | (0.9928-1.3156) | 1.225 | (1.101-1.361) |
| 156 | 1.399 | (1.0179-1.9228) | 1.143 | (0.9919-1.3173) | 1.226 | (1.1012-1.3632) |
